# Victim framing shapes attitudes across diverse contexts

**DOI:** 10.1371/journal.pone.0351416

**Published:** 2026-06-12

**Authors:** Stephen J. Flusberg, Asher Donnelly, J. D. Jarolimek, Esmé Nix, Lili B. Davis, Boshang Yin, Lindsey Anderson, Dylan Ciolfi, Kevin J. Holmes

**Affiliations:** 1 Department of Cognitive Science, Vassar College, Poughkeepsie, New York, United States of America; 2 Department of Psychology, Reed College, Portland, Oregon, United States of America; 3 Department of Psychology, SUNY Purchase College, Purchase, New York, United States of America; PLOS: Public Library of Science, UNITED KINGDOM OF GREAT BRITAIN AND NORTHERN IRELAND

## Abstract

A person accused of victimizing others may be described as the “real” victim by their defenders to garner empathy and mitigate blame. Recent research shows that this rhetorical strategy, known as “victim framing,” can increase support for a man accused of sexually assaulting a woman. Little is known, however, about its effects in other contexts. Across five experiments (*N* = 2,941), we investigated whether victim framing generalizes beyond prototypical sexual assault cases. Participants read fictionalized news reports where one party was labeled the victim (or neither was) and expressed support for the individuals involved. We found significant framing effects across diverse scenarios: (a) a man accused of sexual assault who self-described as the victim; (b) a woman accused of sexually assaulting a man; (c) same-sex assault allegations involving men or women; (d) a celebrity or stranger accused of physically assaulting his girlfriend; and (e) a police officer who shot an unarmed civilian. As in prior work, only participants who explicitly cited the victim-related language as influencing their evaluations showed robust and reliable framing effects. Multiple observer characteristics (e.g., gender, political ideology) predicted attitudes in expected ways, yet victim framing effects persisted when controlling for these individual differences. Taken together, these findings are consistent with a social-pragmatic account of victim framing: many people treat a victim label as communicating relevant information and adjust their evaluations accordingly, while others either do not draw this inference or weigh other information more strongly. Our findings highlight the power and limits of explicit forms of linguistic framing.

## Introduction

Music mogul Sean “Diddy” Combs was arrested on charges of racketeering and sex trafficking in September 2024, and lurid details about his alleged crimes spread rapidly on social media. Combs’ lawyers responded quickly, arguing in a court filing that he was the true victim, the target of false “inflammatory statements” and “outlandish conspiracy theories” [1]. This rhetorical move—where the alleged perpetrator of a crime is cast as the real victim in an effort to garner support and mitigate blame—is known as “victim framing” [2]. Although victims may be judged negatively in some contexts, contemporary social trends have elevated victimhood as a moral status, with victims typically perceived as more virtuous than nonvictims [3,4]. Combs was eventually found guilty of two counts of transportation for the purposes of prostitution, but he was acquitted of the most serious charges.

Victim framing is one element in a broader communication strategy commonly deployed by abusers known as DARVO: Deny, Attack, and Reverse Victim and Offender [5,6]. People called out for misconduct often deny they have done anything wrong, attack the person who accused them, and reframe their accuser as the true offender. The goal is to confuse or gaslight the actual victim, undermine their credibility, and help the perpetrator avoid accountability. Research suggests that DARVO may be effective at shaping attitudes: exposure to DARVO tactics is associated with increased self-blame by victims [7] and reduced perceptions of victim believability by observers [8]. Fortunately, cultivating awareness of DARVO has been shown to diminish its impact [8].

Although victim framing is a key component of DARVO, it can also be deployed independently of the other elements, including by supporters of the alleged wrongdoer. A recent set of studies investigated the impact of explicit third-party victim framing on support for men accused of sexual assault [2]. In the first three experiments, participants read a fictionalized news report about an alleged instance of sexual assault that took place on a college campus. The accuser had come forward with the allegations several months after the incident, and the alleged perpetrator denied any wrongdoing. Either the accuser was labeled the victim of assault (*assault victim* condition), the alleged perpetrator was labeled the victim of false allegations (*allegation victim* condition), or neither individual was described using victim-related language (*baseline* condition). Victim framing was instantiated in both the headline of the report (e.g., “Victim of Sexual Assault Allegations Faces Long Road Ahead”) and in a quote from a friend embedded in the article (e.g., “‘David is a victim of false allegations who deserves to be believed,’ said a close friend of both students”). In a fourth experiment, the report was modified so it described the real-world allegations of sexual assault levied by Dr. Christine Blasey Ford against Justice Brett Kavanaugh. These allegations infamously emerged during Kavanaugh’s Senate confirmation hearing to be seated on the United States Supreme Court in 2018.

After reading the report, participants answered several questions assessing their level of support for the two protagonists in the story. In three of the studies, they also indicated which part of the report was most influential in forming their evaluations by copying and pasting the relevant language into a text box. The results showed that victim framing was generally effective: relative to the baseline condition, people tended to express more support for whomever was labeled the victim. However, this “victim framing effect” was only robust and reliable for those who cited the victim-related language as influencing their judgments. Participants who copied some other part of the text tended to express the same support for the protagonists as those in the baseline condition. The one exception was a *reverse* victim framing effect for participants who did *not* cite the victim-related language in the condition where Brett Kavanaugh was framed as a victim of false allegations. That is, these participants—who were more ideologically liberal and accepted fewer rape myths than most other participants—tended to support Kavanaugh *less* when he was labeled the victim.

Taken together, these findings are consistent with a social-pragmatic account of victim framing. Some people—namely, those who cited the victim-related language as influential—seemed to infer that the victim label was included in the report for a reason: to indicate that a specific individual deserved their support. They then incorporated this information into their evaluations of the parties involved in the assault case. Other people—those who cited factual elements of the case rather than the victim-related language—either failed to draw this pragmatic inference, weighed other information more strongly in their evaluations, or considered and rejected the implications of the victim framing. That would explain why participants who were ideologically opposed to Brett Kavanaugh and rejected common rape myths expressed even *less* support for the judge when he was framed as the victim. This account is grounded in pragmatic theories of language comprehension [9,10] as well as recent work on the role of pragmatic inference in linguistic framing [11], though additional mechanisms may be at work here as well (e.g., dyadic moral reasoning; [2]).

That said, the extent to which explicit victim framing of this sort shapes attitudes in other contexts remains unknown. If the social-pragmatic account is correct, victim framing should generalize to any ambiguous situation involving alleged wrongdoing where people draw inferences about the individuals involved from linguistic cues. On the other hand, it is possible that victim framing is only effective for certain familiar scenarios—like a heteronormative sexual assault case [2]—where people have well-defined cultural scripts or mental models that guide how they make sense of the situation. In less familiar contexts, people may rely more heavily on the specific details of the case or their own moral reasoning to form judgments, and may therefore be less susceptible to victim-related language.

To investigate these possibilities, we designed a series of five experiments that systematically varied key features of the scenario in which victim framing was deployed. Participants read a fictionalized news report that described serious allegations of wrongdoing. Three experiments focused on allegations of sexual assault, as this is a domain where victim framing often appears in public discourse and where the original effects were demonstrated [2]. Within these experiments, we varied the source of the victim label (third party vs. the accused person themselves, Experiment 1) and the gender and sexual orientation of the individuals involved (a woman accused of sexually assaulting a man, in Experiment 2; a same-sex assault involving men or women, in Experiment 3). Experiment 4 examined a different type of misconduct—accusations of physical domestic violence—and varied the social status of the alleged perpetrator (celebrity vs. stranger). In Experiment 5, we extended the paradigm to a fundamentally different context: a police officer shooting an unarmed civilian where the facts of the incident were not under dispute and the protagonists’ names evoked the racial dynamics typical of such cases in the United States. In each scenario, one of the two protagonists was framed as the victim, or else neither character was described using victim-related language. Participants expressed their support for the individuals involved, indicated which part of the report was most influential in forming their evaluations, and responded to several individual differences and demographics measures. This design allowed us to assess whether victim framing generalizes beyond the prototypical sexual assault case, whether it depends on the gender dynamics of the scenario, whether celebrity status moderates its impact, and whether it operates in racially charged contexts of police violence.

Methods and analysis plans were preregistered for all experiments on AsPredicted.org, and are available along with study materials and data on the Open Science Framework (OSF). All experiments were approved by the Institutional Review Boards (IRBs) at the institutions where data collection took place: the Reed College IRB (Experiments 1 and 5); the SUNY Purchase College IRB (Experiments 2 and 4); and the Vassar College IRB (Experiment 3). Informed consent was obtained from all participants via a digital consent form presented at the beginning of each experiment, and all procedures were conducted in accordance with the Declaration of Helsinki.

## Methods

### Participants

We aimed to recruit a preregistered sample of ~100 participants per condition in each experiment (27 total conditions across five experiments; total *N* sampled = 3,057; total *N* analyzed = 2,941), which is consistent with prior studies of victim framing and related research [2,12,13]. Participants who did not complete all measures or who failed an attention check question were removed from analysis or prevented from completing the experiment. Participants were recruited online from one of three crowdsourcing platforms, depending on the experiment: Amazon’s Mechanical Turk (recruited via CloudResearch), Prolific, or CloudResearch Connect. We only recruited people who were at least 18 years old and located in the United States. Data collection for this project began on January 8, 2021, and was completed on November 22, 2024. For complete study demographics, see Table 1.

**Table 1:** Demographic data for all experiments.

	Experiment 1	Experiment 2	Experiment 3	Experiment 4	Experiment 5
Participant pool	MTurk	MTurk	Prolific	CloudResearch Connect	MTurk
Time of data collection	Jan 2021	April 2021	Nov 2024	March 2023	Feb 2021
*N* (analyzed)	600	602	558	577	604
**Gender**					
Female	57.3%	49.3%	63.1%	49%	58.4%
Male	41.8%	49.7%	34.9%	50%	39.9%
Other	0.9%	1%	2.0%	1%	1.7%
Mean age (SD)	39.6 (12.8)	40.7 (12.8)	38.4 (12.7)	40.2 (12.1)	42.8 (13.6)
**Race/ethnicity**					
Asian/Asian Amer	14.7%	9%	5.7%	8.5%	6%
Black/African Amer	6.7%	7.6%	17%	10.2%	9.3%
Hispanic/Latino	4.8%	6.1%	3.2%	4.5%	5.5%
Other/Multiracial	5.7%	3.7%	9.9%	6.4%	4%
White/Caucasian	68.2%	73.6%	64.2%	70.4%	75.3%
**Political affiliation**					
Democrat	43.8%	48%	43.2%	52.9%	47.4%
Republican	26.5%	24.1%	23.7%	24.1%	24.5%
Independent	29.7%	27.9%	33.2%	23.1%	28.1%

### General design and procedure

All experiments were created using Qualtrics online survey software and followed the same general procedure, described below. Experiment-specific materials and any deviations from this shared procedure are described in subsequent sections.

#### Attention check.

After signing the digital consent form, participants completed an attention check question (e.g., “...*in order to demonstrate that you are a participant who reads the study instructions carefully and thoroughly, you need to check the option ‘Other’ below and enter the number 6 in the text box of this option…*”). Participants who failed this attention check were either prevented from completing the experiment (Experiments 1, 2, 5) or had their data removed from analysis (Experiments 3, 4).

#### Stimuli.

After the attention check, participants were randomly assigned to read one of a set of similar fictionalized news reports about an alleged sexual assault (Experiments 1, 2, and 3), physical assault (Experiment 4), or incident of police violence (Experiment 5). In each report, either the alleged perpetrator of misconduct (Experiments 1–4: *allegation victim* condition; Experiment 5: *police victim* condition) or the accuser (Experiments 1–4: *assault victim* condition; Experiment 5: *civilian victim* condition) was framed as the victim, or else neither party was framed as the victim (*baseline* condition). The victim-framing language was presented in both the headline and the main text, as in past work on victim framing [2]. Participants were prevented from advancing to the next screen for either 15 or 25 seconds, depending on which report they were assigned to read. The baseline condition served as a no-label control since neither individual was described using victim-related language. Importantly, however, participants in this condition were not in a psychologically neutral state: they brought their own cultural scripts, prior beliefs, and moral convictions to bear on the same scenario as those in the victim framing conditions. The baseline condition therefore provides a reference point for evaluating the incremental impact of explicit victim framing in shaping judgments in these cases.

#### Support measure.

On the following screen, participants responded to a series of statements assessing their level of support for the two protagonists in the report. Using a scale from 0 (*none/not at all*) to 6 (*a lot/very*), participants in Experiments 1–4 indicated how much *empathy* they had for each character, how *believable* each of them was, how much *harm* each had experienced as a result of the incident, and how *responsible* each was for the incident. Which character participants assessed first in each pair of statements was counterbalanced across participants in each study. In Experiment 5, participants responded to the same *empathy* and *responsibility* statements, but the *harm* and *believability* statements were removed because of the unambiguous nature of the shooting incident described in the report. Instead, participants indicated how much *emotional suffering* was endured by each character. For every participant, we computed a mean *support* score for each protagonist by averaging their responses to these three or four questions (reverse-coding *responsibility* scores; Cronbach’s α = .70–.85 across all protagonists and experiments). Analyses of these composite support scores are reported in the Results and discussion section below (see S1 Text for analyses of the disaggregated individual components).

Following the *support* measure, participants in all but one study (Experiment 1) provided their general opinion of both protagonists using a free response text box. In Experiments 2 and 5, participants were also asked to indicate whether the situation described in the article reminded them of any real-life events, and to specify if yes. We do not report on these *general opinion* or *reminded of real life event* data in the present paper, but the raw data are available on the OSF.

#### Citing measure.

On the next screen, participants were shown the report they had read and were instructed to copy and paste the part of the article that was most influential in forming their evaluation of the individuals in the report. They were also provided with another free response text box and asked if there was any other information that contributed to their evaluations. We used automated text coding in Microsoft Excel to determine whether participants in the victim framing conditions explicitly relied on the victim-related language in forming their judgments. Those who included victim-related language from the report in any of their rationales (i.e., the words “victim,” “traumatized,” or “survive,” or cognates with the same root) we refer to as *citers*, while *nonciters* were those who copied or mentioned any other part of the story. As in prior work on victim framing [2], we used this citer/nonciter distinction as an indirect marker of pragmatic sensitivity to the victim framing language, though it does not directly assess the underlying inferential process.

#### Additional measures.

Participants were then asked to complete several personal experience and individual difference measures, which varied across experiments in accordance with the specific topic under consideration (Table 2). These measures enabled us to assess whether preexisting attitudes predict support for the protagonists in expected ways, and whether victim framing effects persist above and beyond such attitudes, though these analyses were preregistered as exploratory (see S1 Text). Finally, participants answered a set of basic demographic questions and were debriefed and thanked for their time.

**Table 2:** Additional individual differences measures completed by participants in each experiment.

Measure	Citation	Exp.	Description	Response Mode	Sample Item
Acceptance of Interpersonal Violence Scale (AIV)	[14]	2, 3	A 6-item measure that assesses acceptance of the belief that force and coercion are viable means to gain compliance in intimate relationships	5-point Likert (1 = Strongly disagree, 5 = Strongly agree)	“Sometimes the only way a man can get a cold woman turned on is to use force”
Attitudes Toward the Criminal Legal System Scale (ATCLS)	[15]	5	A 38-item measure that assesses perceptions of juries, punishment, laws, defense attorneys, judges, police, and prosecutors	7-point Likert (1 = Strongly disagree, 7 = Strongly agree)	“Juries often base decisions on their prejudices instead of facts”
Attitudes Toward Lesbians and Gay Men (ATLG)	[16]	3	A 10-item measure of people’s attitudes toward gay men and lesbians	5-point Likert (1 = Strongly disagree, 5 = Strongly agree)	“Sex between two men is just plain wrong”
Attitudes Towards Police Legitimacy Scale (ATPLS)	[17]	5	A 34-item measure that assesses feelings of police bias, quality of interpersonal treatment, trustworthiness, motivation, quality and organizational integrity, community, and normative alignment	7-point Likert (1 = Strongly disagree, 7 = Strongly agree)	“Police officers usually make fair decisions when enforcing laws”
Experience with Sexual Assault	[14]	2, 3	A 5-item measure of one’s personal experience with sexual assault	Yes or No and Free response	“Have you ever had anyone force sex on you against your will?”
Modified Illinois Rape Myth Acceptance Scale (IRMA)	[18]	2, 3	A 19-item measure of one’s attitudes and beliefs about sexual assault	5-point Likert (1 = Strongly disagree, 5 = Strongly agree)	“If a woman is raped while she is drunk, she is at least somewhat responsible for letting things get out of control”
Short Dark Triad Scale (SD3)	[19]	1	A 27-item self-report measure of three socially aversive personality traits— Machiavellianism, psychopathy, and narcissism—using 9 items per trait	5-point Likert (1 = Strongly disagree, 5 = Strongly agree)	“I insist on getting the respect I deserve”
Social Desirability Scale (SDS)	[20]	1	A 10-item measure that assesses the tendency to respond in a socially acceptable manner rather than truthfully	True or False	“I have never deliberately said something that hurt someone’s feelings”

### Experiment 1

In many cases of misconduct, it is the perpetrator who self-describes as a victim in order to avoid accountability, as research on DARVO has demonstrated [5,6]. However, previous research on victim framing has focused on cases in which the framer is a third-party supporter [2; but see 8]. On one hand, people tend to assume that others are honest and informative in their communications [9,10,21], suggesting victim framing could still be effective in this instance. On the other hand, people may assume that self-serving statements are not truthful in situations where an alleged assailant is motivated to present themselves in a positive light [21]. In this case, self-victim framing might be less effective than other-victim framing. We tested this possibility in Experiment 1, exploring whether the effect of victim framing in sexual assault cases extends to individuals self-describing as victims. We used a 3 (victim frame: assault victim vs. allegation victim vs. baseline) × 2 (framer: self vs. other) between-subjects design. The news reports in this study described an alleged sexual assault in which a male college student assaulted a female student at a fraternity party, adapting the vignette from earlier studies of victim framing [2]. See Table 3 for stimulus paragraphs.

**Table 3:** News report stimuli used in Experiment 1 (self-framing vs. other-framing scenarios).

Report section	Frame		
	Assault Victim	Allegation Victim	Baseline
Headline (participants read either the self-framed or other-framed headline within each victim frame condition)	*Self Frame*		
	“I Am a Victim of Sexual Assault,” Claims Student May 4, 2020	“I Am a Victim of False Allegations,” Claims Student Accused of Sexual Assault May 4, 2020	Students Face Sexual Assault Investigation May 4, 2020
	*Other Frame*		
	Victim of Sexual Assault Faces Investigation May 4, 2020	Victim of Sexual Assault Allegations Faces Investigation May 4, 2020	Students Face Sexual Assault Investigation May 4, 2020
Description of alleged event (identical for all frame conditions)	Emma Sawyer, a student at Livingston University, filed a complaint against fellow student David Bradley, alleging that Bradley sexually assaulted her at a Halloween party hosted by the Sigma Chi fraternity last fall. In the complaint, Sawyer claimed that Bradley lured her to a private bedroom in the fraternity house and proceeded to remove her clothing, pin her to a bed, and force himself onto her despite her protestations. University authorities are investigating Sawyer’s allegations, which Bradley has firmly denied. Bradley also questioned why the allegations had not surfaced until six months after the purported incident and noted that he and Sawyer were both intoxicated on the night in question.		
Framing section	*Self Frame*		
	Reactions on campus are polarized, but Sawyer has not wavered. “I am a victim of sexual assault and I deserve to be believed,” said Sawyer. “I have been traumatized by this experience. The investigation ahead is going to be long and grueling, but I hope I’ll survive it.”	Reactions on campus are polarized, but Bradley has maintained his innocence. “I am a victim of false allegations and I deserve to be believed,” said Bradley. “I have been traumatized by this experience. The investigation ahead is going to be long and grueling, but I hope I’ll survive it.”	Reactions on campus are polarized, but Sawyer and Bradley have both maintained their accounts. “I should be allowed to tell my side of the story,” said Bradley. Added Sawyer, “This experience has been challenging for me. The investigation ahead is going to be long and grueling, but I know the truth will come out.”
	*Other Frame*		
	Reactions on campus are polarized, but friends have rallied around Sawyer. “Emma is a victim of sexual assault who deserves to be believed,” said a close friend of both students. “She has been traumatized by this experience. The investigation ahead is going to be long and grueling, but I know she will survive it.”	Reactions on campus are polarized, but friends have rallied around Bradley. “David is a victim of false allegations who deserves to be believed,” said a close friend of both students. “He has been traumatized by this experience. The investigation ahead is going to be long and grueling, but I know he will survive it.”	Reactions on campus are polarized, but friends have rallied around both Sawyer and Bradley. “Emma and David should each be allowed to tell their side of the story,” said a close friend of both students. Added another, “This experience has been challenging for both of them. The investigation ahead is going to be long and grueling, but I know the truth will come out.”

**Note:** Participants were randomly assigned to one of the three victim frame conditions and to either the *self* or *other* frame condition. Each participant read one of the six headlines, followed by the general description of the alleged event. The end of the paragraph was framed in a way that was consistent with the headline, for a total of six unique reports (two per victim frame condition).

After reading the news report and completing the *support* measure, participants assessed both protagonists in the story on the socially aversive “Dark Triad” personality traits of Machiavellianism (strategic manipulation), psychopathy (callousness and lack of empathy), and narcissism (grandiosity and entitlement). We incorporated this measure because past research has shown that people who cast themselves as victims tend to exhibit more Dark Triad traits, on both self-report and behavioral measures [22]. We were therefore interested in whether participants would judge self-victim framing characters as being higher on these traits than other-victim framing characters, and if this would moderate the victim framing effect on support. We adapted the Dirty Dozen scale [23], a 12-item self-report measure of Dark Triad traits. Participants rated both protagonists in the story on four items associated with Machiavellianism (e.g., “David tends to manipulate others to get his way”), four items associated with psychopathy (e.g., “Emma tends to lack remorse”), and four items associated with narcissism (e.g., “David tends to want others to admire him”), using a 5-point Likert scale ranging from 1 (strongly disagree) to 5 (strongly agree). We created composite Dark Triad perception scores for both Emma and David by averaging responses to the 12 items for each character (Cronbach’s α = .97 and  .96, respectively).

Additional measures completed after the *citing* measure included the self-report Short Dark Triad Scale (SD3) and the Social Desirability Scale (SDS; see Table 2).

### Experiment 2

Previous research has found that perceptions of victimhood are influenced by gender stereotypes [24]. People typically view men as more agentic, violent, and aggressive, while women are seen as more passive and vulnerable to harm. Although most incidents of male rape are perpetrated by women, this crime often goes unreported because it clashes with stereotypes and cultural models of masculinity and sexual power [25]. It is plausible, therefore, that cultural gender narratives could override any effect of framing a male accuser as a victim of sexual assault. We tested this possibility in Experiment 2.

Participants were randomly assigned to read one of six fictionalized news reports about an incident at a sorority party in which a woman allegedly sexually assaulted a man. We used a 3 (frame: assault victim vs. allegation victim vs. baseline) × 2 (detail: rich vs. sparse) between-subjects design. We varied whether the alleged victim, the alleged perpetrator, or neither student was labeled the victim, and whether or not the news article provided a detailed account of the alleged assault (see Table 4 for complete stimuli). Additional measures included self-reports of personal experience with sexual assault and acceptance of interpersonal violence, and the modified Illinois Rape Myth Acceptance Scale (see Table 2).

**Table 4:** News report stimuli used in Experiment 2 (male accuser scenario).

Report section	Frame		
	Assault Victim	Allegation Victim	Baseline
Headline	Victim of Sexual Assault Faces Long Road Ahead (May 4, 2018)	Victim of Sexual Assault Allegations Faces Long Road Ahead (May 4, 2018)	Students Embroiled in Sexual Assault Investigation Face Long Road Ahead (May 4, 2018)
Description of alleged event (participants received the sparse- or rich-detail paragraph)	*Sparse Detail*		
	David Bradley, a student at Livingston University, filed a complaint against fellow student Emma Sawyer, alleging that Sawyer sexually assaulted him at a campus party last fall. University authorities are investigating Bradley’s allegations, which Sawyer has firmly denied.		
	*Rich Detail*		
	David Bradley, a student at Livingston University, filed a complaint against fellow student Emma Sawyer, alleging that Sawyer sexually assaulted him at a Halloween party hosted by the Delta Gamma sorority last fall. In the complaint, Bradley claimed that Sawyer lured him to a private bedroom in the sorority house and proceeded to remove his clothing, pin him to a bed, and force herself onto him despite his protestations. University authorities are investigating Bradley’s allegations, which Sawyer has firmly denied. Sawyer also questioned why the allegations had not surfaced until six months after the purported incident and noted that she and Bradley were both intoxicated on the night in question.		
Framing section	Reactions on campus are polarized, but friends have rallied around Bradley. “David is a victim of sexual assault who deserves to be believed,” said a close friend of both students. “He has been traumatized by this experience. The investigation ahead is going to be long and grueling, but I know he will survive it.”	Reactions on campus are polarized, but friends have rallied around Sawyer. “Emma is a victim of false allegations who deserves to be believed,” said a close friend of both students. “She has been traumatized by this experience. The investigation ahead is going to be long and grueling, but I know she will survive it.”	Reactions on campus are polarized, but friends have rallied around both Bradley and Sawyer. “David and Emma should each be allowed to tell their side of the story,” said a close friend of both students. “This experience has been challenging for both of them. The investigation ahead is going to be long and grueling, but I know the truth will come out.”

**Note:** Participants read one of the three headlines, followed by either the sparse- or rich-detail paragraph. The end of the paragraph was framed in a way that was consistent with the headline, for a total of six unique reports.

### Experiment 3

Most media coverage of sexual assault concerns heterosexual and heteronormative cases, as in prior work on victim framing [2,25]. To examine the effects of victim framing in cases involving individuals of other sexual orientations, the news report in Experiment 3 described an alleged sexual assault that took place between two people of the same gender at a dorm hall party. Similar to Experiment 2, the lack of cultural narratives about same-sex assault may render victim framing less impactful in this situation. Victim framing might also differentially impact judgments about cases involving two men versus two women due to gender stereotypes about moral agency [24]. Alternatively, victim framing may generalize to all assault cases, regardless of the genders or sexual orientations of the alleged perpetrator and the accuser. To adjudicate between these possibilities, we used a 3 (frame: assault victim vs. allegation victim vs. baseline) × 2 (gender: male vs. female) between-subjects design in Experiment 3, where we varied the victim frame and whether the alleged assault involved two women or two men. Each scenario included a detailed account of the event (see Table 5). Additional measures included self-reports of personal experience with sexual assault and acceptance of interpersonal violence, the modified Illinois Rape Myth Acceptance Scale, and the Attitudes Toward Lesbians and Gay Men Scale (see Table 2).

**Table 5:** News report stimuli used in Experiment 3 (same-sex assault scenarios).

Report section	Frame		
	Assault Victim	Allegation Victim	Baseline
Headline	Victim of Sexual Assault Faces Long Road Ahead (October 4, 2024)	Victim of Sexual Assault Allegations Faces Long Road Ahead (October 4, 2024)	Students Embroiled in Sexual Assault Investigation Face Long Road Ahead (October 4, 2024)
Description of alleged event (identical for all frame conditions)	[Lila/Leo] Sawyer, a sophomore on the [women’s/men’s] tennis team at Livingston University, filed a complaint against [her/his] teammate [Vivian/Julian] Bradley, alleging that [Ms./Mr.] Bradley sexually assaulted [her/him] at a dorm hall party last spring. In the complaint, [Ms./Mr.] Sawyer claimed that [Ms./Mr.] Bradley lured [her/him] to a dorm room and proceeded to remove [her/his] clothing, pin [her/him] to a bed, and force [her/him]self onto [Ms./Mr.] Sawyer despite [her/his] protestations. University authorities are investigating [Ms./Mr.] Sawyer’s allegations, which [Ms./Mr.] Bradley has firmly denied. [Ms./Mr.] Bradley also questioned why the allegations had not surfaced until six months after the purported incident and noted that [she/he] and [Ms./Mr.] Sawyer were both intoxicated on the night in question.		
Framing section	Reactions on campus are polarized, but friends have rallied around [Ms./Mr.] Sawyer. “[Lila/Leo] is a victim of sexual assault who deserves to be believed,” said a close friend of both students. “[She/He] has been traumatized by this experience. The investigation ahead is going to be long and grueling, but I know [she/he] will survive it.”	Reactions on campus are polarized, but friends have rallied around [Ms./Mr.] Bradley. “[Vivian/Julian] is a victim of false allegations who deserves to be believed,” said a close friend of both students. “[She/He] has been traumatized by this experience. The investigation ahead is going to be long and grueling, but I know [she/he] will survive it.”	Reactions on campus are polarized, but friends have rallied around both [Ms./Mr.] Sawyer and [Ms./Mr.] Bradley. “[Lila/Leo] and [Vivian/Julian] should each be allowed to tell their side of the story,” said a close friend of both students. “This experience has been challenging for both of them. The investigation ahead is going to be long and grueling, but I know the truth will come out.”

**Note:** Participants read one of the three headlines, followed by the general description of the alleged event. The end of the paragraph was framed in a way that was consistent with the headline, for a total of six unique reports.

### Experiment 4

All prior studies of victim framing involved sexual assault allegations—a domain that is frequently presented in the media using competing victim narratives [2]. However, victim framing can be deployed when there are allegations of misconduct of any kind. In Experiment 4, the news report described an alleged incident of physical domestic abuse. Such allegations are especially likely to receive public attention when they involve prominent, well-known individuals. At the same time, people may evaluate celebrities accused of wrongdoing differently from anonymous strangers. Research has shown that familiarity, exposure, and attractiveness all influence people’s social judgments [26–29], and people may be more forgiving of celebrities with whom they have strong parasocial relationships [30–32]. In this experiment, we tested whether the effects of victim framing extend to a physical assault scenario involving a familiar celebrity. We used a 3 (victim frame: assault victim vs. allegation victim vs. baseline) × 2 (celebrity status: stranger vs. celebrity) between-subjects design. In the news reports, we varied both the victim frame and whether the alleged assault was committed by a local plumber or by award-winning actor Nicolas Cage. The scenario involving Cage was adapted from a real news story involving allegations of domestic abuse against the actor by a former girlfriend [33], though he was never prosecuted for the incident (see Table 6).

**Table 6:** News report stimuli used in Experiment 4 (celebrity assault scenario).

Report section	Frame		
	Assault Victim	Allegation Victim	Baseline
Headline (participants read either the celebrity or stranger headline within each frame condition)	*Celebrity*		
	Victim of Celebrity Assault Faces Long Road Ahead (September 20, 2018; Vienna, Austria)	Celebrity Victim of Assault Allegations Faces Long Road Ahead (September 20, 2018; Vienna, Austria)	Former Lovers Embroiled in Assault Allegations Face Long Road Ahead (September 20, 2018; Vienna, Austria)
	*Stranger*		
	Victim of Assault Faces Long Road Ahead (September 20, 2018; Holland, MI)	Victim of Assault Allegations Faces Long Road Ahead (September 20, 2018; Holland, MI)	Former Lovers Embroiled in Assault Allegations Face Long Road Ahead (September 20, 2018; Holland, MI)
Description of alleged event (identical for all frame conditions)	[Vicky/Sarah] Park, former girlfriend of [American actor Nicolas Cage/Nicolas Hatch], filed a complaint against the [National Treasure star/local plumber], alleging that [Cage/Hatch] physically assaulted her last month at the [Slash Film Festival in Vienna/Fall Film Festival in town]. According to Park, [the actor/Hatch] was “severely intoxicated” at the time. Court documents filed in the case reference a history of alcohol addiction and depression, but do not go into more details about the alleged abuse.		
Framing section	Reactions [online/in the community] are polarized, but friends have rallied around Park. “[Vicky/Sarah] is a victim of assault who deserves to be believed,” said a close friend of the former couple. “She has been traumatized by this experience. The investigation ahead is going to be long and grueling, but I know she will survive it.”	Reactions [online/in the community] are polarized, but friends have rallied around [Cage/Hatch]. “Nick is a victim of false allegations who deserves to be believed,” said a close friend of the former couple. “He has been traumatized by this experience. The investigation ahead is going to be long and grueling, but I know he will survive it.”	Reactions [online/in the community] are polarized, but friends have rallied around both Park and [Cage/Hatch]. “[Vicky/ Sarah] and Nick should each be allowed to tell their side of the story,” said a close friend of the former couple. “This experience has been challenging for both of them. The investigation ahead is going to be long and grueling, but I know the truth will come out.”

**Notes:** Participants read one of the six headlines, followed by the general description of the event. The end of the paragraph was framed in a way that was consistent with the headline, for a total of six unique reports.

### Experiment 5

Previous studies of victim framing all involved cases of alleged interpersonal violence where there was plausible deniability for the accused perpetrator. This maximized the odds of eliciting victim framing effects, as the facts of the matter were under dispute and victimhood was equivocal. However, victim framing can be deployed even when the facts of the case are well known, as long as there are competing ways of evaluating the event that license different moral judgments about those involved. For example, a police officer who shoots an unarmed civilian might claim they genuinely felt threatened and were therefore the victim of an aggressive individual who did not respect the officer’s warning.

It is unclear whether victim framing would work in the same way in this scenario as in cases with arguable facts. For one thing, there are clear differences in power, authority, and legal obligations between police officers and civilians. People’s knowledge of the legal system might make it more difficult to “typecast” police officers in the victim/patient role because these institutions are viewed as legitimate systems of authority [34,35]. It is possible, therefore, that victim framing may be less effective in this context. Alternatively, because of their privileged position in society, even violent actions perpetrated by police officers might be perceived as morally justifiable, which could increase the efficacy of victim framing [35,36]. Finally, pre-existing attitudes toward the police and the legal system could be especially likely to moderate the impact of victim framing in this context [35].

Experiment 5 was designed to investigate these possibilities. Participants read a news report about an incident that took place in a supermarket parking lot in which a police officer shot a civilian in the leg after the civilian failed to comply with instructions to return to his car. There were three between-subject conditions: either the civilian was labeled the victim of police violence, the police officer was labeled the victim of a lack of respect, or neither party was framed as the victim (baseline). The names of the protagonists—Jamal Smith for the civilian and John O’Neil for the police officer—were selected to evoke the racial dynamics commonly associated with highly publicized incidents of police violence in the United States, where media coverage has focused on cases of White officers using force against Black civilians. However, we did not explicitly manipulate or directly measure participants’ racial attributions. Therefore, race is likely an *implicit* feature of how participants construed the scenario. This is another factor that could potentially overshadow the effects of victim framing, by making a different contentious cultural script more salient. See Table 7 for stimuli.

**Table 7:** News report stimuli used in Experiment 5 (police violence scenario).

Report section	Frame		
	Civilian Victim	Police Victim	Baseline
Headline	Livingston Resident is a Victim of Police Violence (April 10, 2019)	Livingston Police Officer is a Victim of Enforcing the Law (April 10, 2019)	Livingston Police Officer and Resident Await Investigation (April 10, 2019)
Description of event (identical for all frame conditions)	A violent encounter last night between a Livingston police officer and a civilian ended with a gunshot wound, the civilian in the hospital, and outrage in the community. At approximately 10:30 p.m. on April 9, Officer John O’Neil responded to reports of a disturbance in a Safeway parking lot in northeast Livingston, a hub for local gang activity. Upon O’Neil’s arrival at the scene, local resident Jamal Smith exited a parked car and did not respond to O’Neil’s order to return to his vehicle. O’Neil proceeded to shoot Smith in the leg. Paramedics rushed Smith to the hospital, where he is in stable condition and is expected to recover as he awaits legal proceedings. An investigation of the incident is underway. In the meantime, Officer O’Neil has been assigned to desk duty, has been stripped of his firearm, and faces calls for his termination from the force.		
Framing section	Said a Safeway employee who witnessed the incident, “I saw the whole situation go down. Jamal is yet another victim of the police using excessive force.” De’ja Smith, Jamal’s wife, also defended her husband. “Jamal is the real victim of this altercation. I pray that the law will bring him justice and that there will be no more trauma.”	Said a Safeway employee who witnessed the incident, “I saw the whole situation go down. Officer O’Neil is yet another victim of people not respecting cops.” Susan O’Neil, the officer’s wife, also defended her husband. “John is the real victim of this altercation. I pray that the law will bring him justice and that there will be no more trauma.”	Said a Safeway employee who witnessed the incident, “I saw the whole situation go down. I pray that the law will bring both men justice and that there will be no more trauma.”

**Note:** Each participant read one of the three headlines, followed by the general description of the event. The end of the paragraph was framed in a way that was consistent with the headline, for a total of three unique reports.

Notably, we ran a pilot version of this experiment that also manipulated the inclusion of information on “qualified immunity,” a policy that protects law enforcement from civil lawsuits. Although we observed a similar pattern of results in this study, the stimuli were not well balanced, which led us to refine and simplify the study design. See S1 Text for a detailed account of the pilot study.

After the *support* questions, participants responded to two questions assessing their beliefs about the aftermath of the violent incident. First, they were asked “How do you think the safety of the Livingston community has changed as a result of the incident?” They responded using a Likert scale ranging from 0 (much less safe) to 6 (much more safe). They were then asked “In whose favor do you think the investigation will be resolved?” and selected a response from a multiple choice list: Jamal Smith, John O’Neil, or Not sure (please explain). We do not report on these data here, but they are available on the OSF. After the *citing* measure, participants completed two additional measures (see Table 2): the Attitudes Towards Police Legitimacy Scale (ATPLS) and the Attitudes Towards the Criminal Legal System Scale (ATCLS).

## Results and discussion

### Overall approach

For each experiment, we conducted a series of analyses using JASP 0.95.4 [37], an open-source statistical analysis program. The *preregistered* analyses mirrored those conducted in prior work on victim framing [2], except where otherwise noted below. Specifically, we conducted (1) analyses of variance (ANOVAs)—standard statistical tests for comparing group means—to assess the overall impact of the victim framing manipulation and any additional independent variable in a given experiment on *support* scores for each of the protagonists described in the news report (plus *Dark Triad* scores in Experiment 1), and (2) ANOVAs to assess the interaction between frame and whether or not a participant cited the victim-related language in the report as influencing their evaluations (i.e., *citer status*: citers vs. nonciters). The baseline condition was excluded from the latter analyses because the baseline stimuli did not contain victim-related language.

There were minor deviations from the above analyses in the preregistered analysis plans for Experiments 1 and 5, but the analyses we conducted and report below are consistent with prior work [2] and the other experiments in this series. Specifically, in Experiment 1 we preregistered a combination of (1) and (2) above, as opposed to separate analyses, and in Experiment 5 we omitted analysis (1) above (see OSF for preregistration details).

In addition to these preregistered analyses, we conducted a series of *exploratory* analyses in each study examining whether any of the additional measures and demographic variables predicted support for the protagonists, whether the impact of victim framing remained when controlling for these variables, and whether citers and nonciters differed in meaningful ways on these measures. These exploratory analyses are reported in full in the S1 Text. Below, we report the results of the preregistered analyses.

### Experiment 1

In this experiment, we tested whether “self-victim” (versus “other-victim”) framing affected support for and Dark Triad perceptions of protagonists in a news report about an alleged sexual assault at a college party. See Table 8 for means and standard errors (SEs).

**Table 8:** Mean support and Dark Triad scores by frame, framer, and citer status in Experiment 1.

Measure	Framer	Protagonist	Frame		
			*Assault Victim*	*Allegation Victim*	*Baseline*
Support	Self	Accuser (Emma)	4.47 (0.12)	4.16 (0.13)	4.47 (0.12)
		Alleged Perpetrator (David)	2.03 (0.14)	2.88 (0.15)	2.30 (0.13)
	Other	Accuser (Emma)	4.28 (0.14)	3.99 (0.14)	4.27 (0.12)
		Alleged Perpetrator (David)	2.34 (0.15)	2.65 (0.14)	2.39 (0.14)
	*Total*	*Accuser (Emma)*	4.37 (0.09)	4.08 (0.09)	4.37 (0.08)
		*Alleged Perpetrator (David)*	2.18 (0.10)	2.77 (0.10)	2.35 (0.09)
Dark Triad	Self	Accuser (Emma)	2.35 (0.10)	2.49 (0.11)	2.32 (0.09)
		Alleged Perpetrator (David)	3.49 (0.09)	3.09 (0.10)	3.35 (0.08)
	Other	Accuser (Emma)	2.37 (0.10)	2.67 (0.09)	2.30 (0.09)
		Alleged Perpetrator (David)	3.35 (0.09)	3.25 (0.09)	3.29 (0.09)
	*Total*	*Accuser (Emma)*	2.36 (0.07)	2.57 (0.07)	2.31 (0.06)
		*Alleged Perpetrator (David)*	3.42 (0.06)	3.17 (0.07)	3.32 (0.06)
**Measure**	**Citer Status**	**Protagonist**	**Frame**		
			*Assault Victim*	*Allegation Victim*	**Contrast**
Support	Citer	Accuser (Emma)	4.79 (0.12)	4.13 (0.15)	**
		Alleged Perpetrator (David)	1.93 (0.16)	2.80 (0.16)	***
	Nonciter	Accuser (Emma)	4.10 (0.12)	4.05 (0.12)	ns
		Alleged Perpetrator (David)	2.34 (0.13)	2.75 (0.13)	*
Dark Triad	Citer	Accuser (Emma)	2.11 (0.10)	2.66 (0.18)	***
		Alleged Perpetrator (David)	3.61 (0.10)	3.24 (0.11)	*
	Nonciter	Accuser (Emma)	2.51 (0.09)	2.53 (0.09)	ns
		Alleged Perpetrator (David)	3.29 (0.08)	3.13 (0.08)	ns

**Note:** Standard errors (SEs) in parentheses; citer status data collapsed across framer conditions; planned contrasts: ***p < .001; **p < .01; *p < .05

#### Support scores: Overall framing effects.

We conducted two 3 (frame: assault victim vs. allegation victim vs. baseline) × 2 (framer: self vs. other) ANOVAs on support scores for each protagonist. There was a main effect of frame on support for both protagonists: Emma: *F*(2, 594) = 3.65, *p* = .027, *η*_*p*_*²* = .012; David: *F*(2, 594) = 9.40, *p* < .001, *η*_*p*_*²* = .031. Support for the accuser (Emma) was lower in the allegation-victim condition than in both the assault-victim and baseline conditions, though these differences were not statistically significant in Bonferroni-corrected post-hoc tests; *t*(594) = 2.36, *p* = .056, *d* = 0.24, and *t*(594) = 2.31, *p* = .063, *d* = 0.23, respec*t*ively. There was no difference in support for Emma between the assault-victim and baseline conditions (*p* > .99). Conversely, support the alleged perpetrator (David) was significantly higher in the allegation-victim condition than in both the assault-victim and baseline conditions; *t*(594) = 4.21, *p* < .001, *d* = 0.42, and *t*(594) = 2.99, *p* = .008, *d* = 0.3, respectively. There was no difference in support for David between the assault-victim and baseline conditions (*p* = .69). There was no main effect of framer on support for either protagonist—Emma: *F*(1, 594) = 3.25, *p* = .072, *η*_*p*_*²* = .005; David: *F*(1, 594) = 0.26, *p* = .61, *η*_*p*_*²* < .001. Nor were there significant interactions between frame and framer on support for either protagonist—Emma: *F*(2, 594) = .01, *p* = .99, *η*_*p*_*²* < .001; David: *F*(2, 594) = 1.9, *p* = .15, *η*_*p*_*²* < .01.

#### Support scores: Citers versus nonciters.

**Fig 1:**
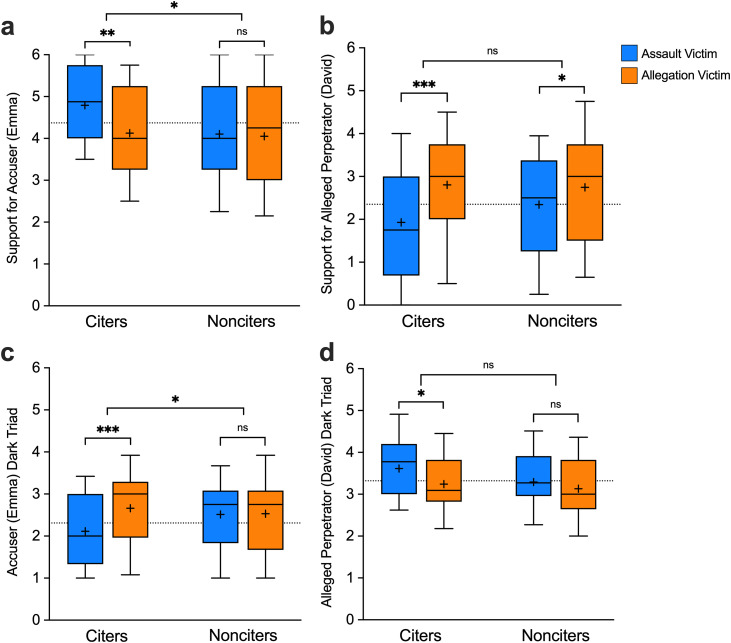
Support/Dark Triad scores for (a/c) the accuser and (b/d) the alleged perpetrator in the assault-victim and allegation-victim conditions of Experiment 1, plotted separately for participants who cited victim-related language as influential (“citers”) and those who did not (“nonciters”). Data are collapsed across framer conditions (self/other). Boxes denote the interquartile range, the horizontal middle line indicates the median, whiskers extend from the 10th to the 90th percentiles, and the plus sign denotes the mean. The dashed line indicates the mean in the baseline condition. ***p < .001; **p < .01; *p < .05. Citers showed the expected framing effect on support and Dark Triad judgments of the accuser and alleged perpetrator alike, while nonciters showed only a small framing effect on support for the alleged perpetrator.

Across the two victim framing conditions, 36.5% of participants included one or more victim-related words in their rationales and were classified as citers. Since there was no effect of framer in the previous analyses, we removed this factor and conducted separate 2 (frame: assault victim vs. allegation victim) × 2 (citer status: citer vs. nonciter) ANOVAs on support for each protagonist. As in our previous analyses, there was a main effect of frame on support for both protagonists, with greater support for Emma in the assault-victim condition and greater support for David in the allegation-victim condition—Emma: *F*(1, 399) = 7.05, *p* = .008, *η*_*p*_*²* = .017; David: *F*(1, 399) = 18.71, *p* < .001, *η*_*p*_*²* = .045. There was also a main effect of citer status on support for Emma, *F*(1, 399) = 8.01, *p* = .005, *η*_*p*_*²* = .02, but not for David, *F*(1, 399) = 1.45, *p* = .23, *η*_*p*_*²* < .01. The former effect was qualified by a significant interaction between frame and citer status on support for Emma, *F*(1, 399) = 5.17, *p* = .024, *η*_*p*_*²* = .013. Planned contrasts revealed that, for citers, support for Emma was significantly higher in the assault-victim condition, where she was framed as the victim, than in the allegation-victim condition, where David was framed as the victim, *t*(399) = 3.09, *p* = .002, *d* = 0.51. For nonciters, there was no difference in support for Emma in the allegation-victim and assault-victim conditions, *t*(399) = .31, *p* = .75, *d* = 0.04 (Fig 1a). There was no significant in*t*eraction between frame and citer status on support for David, *F*(1, 399) = 2.55, *p* = .11, *η*_*p*_*²* < .01, though numeric trends were in the expected direction. The absence of a significant interaction likely reflects the fact that both citers and nonciters showed some sensitivity to the framing of the alleged perpetrator, leaving less room for a divergence to emerge. Consistent with this possibility, exploratory contrasts revealed that both citers and nonciters expressed significantly more support for David in the allegation-victim condition than in the assault-victim condition, though the magnitude of this difference was (nonsignificantly) larger for citers; *t*(399) = 3.71, *p* < .001, *d* = 0.61 vs. *t*(399) = 2.26, *p* = .024, *d* = 0.28 for citers and nonciters, respectively (Fig 1b).

#### Dark Triad scores: Overall framing effects.

Next, we conducted two 3 (frame: assault victim vs. allegation victim vs. baseline) × 2 (framer: self vs. other) ANOVAs on Emma’s and David’s composite *Dark Triad* scores. The results mirrored our findings for support scores. There was a main effect of frame for both protagonists—Emma: *F*(2, 594) = 4.34, *p* = .013, *η*_*p*_^*2*^ = 0.014; David: *F*(2, 594) = 3.88, *p* = .021, *η*_*p*_^*2*^ = .013. Participants perceived the accuser, Emma, as higher on Dark Triad traits in the allegation-victim condition, where David was framed as the victim, than in the baseline and assault-victim conditions. However, only the former difference was statistically significant in Bonferroni-corrected post-hoc tests; *t*(594) = 2.74, *p* = .019, *d* = 0.27, and *t*(594) = 2.29, *p* = .067, *d* = 0.23, respectively. Similarly, participants perceived the alleged perpetrator, David, as lower on Dark Triad traits when he was framed as the victim (allegation-victim condition) than in the assault-victim and baseline conditions. Again, only the former difference was statistically significant in Bonferroni-corrected post-hoc tests; *t*(594) = 2.77, *p* = .018, *d* = 0.28, and *t*(594) = 1.65, *p* = .30, *d* = 0.16, respectively. There was no difference in Dark Triad scores for Emma or David for participants in the assault-victim and baseline conditions (*t*s < 1.2, *p*s > .8). Nor was there a main effect of framer on Dark Triad scores for either protagonist—Emma: *F*(2, 594) = 0.52, *p* = .47, *η*_*p*_^*2*^ < .001; David: *F*(2, 594) = 0.05, *p* = .82, *η*_*p*_^*2*^  < .001. There was also no significant interaction between frame and framer—Emma: *F*(2, 594) = 0.59, *p* = .56, *η*_*p*_^*2*^  < .01; David: *F*(2, 594) = 1.38, *p* = .25, *η*_*p*_^*2*^ < .01.

#### Dark Triad scores: Citers versus nonciters.

We conducted separate 2 (frame: assault victim vs. allegation victim) × 2 (citer status: citer vs. nonciter) ANOVAs on Dark Triad scores for each protagonist. The results mostly mirrored our findings for support scores. As in our previous analyses, there was a main effect of frame on Dark Triad scores for both protagonists, with Emma perceived as higher on Dark Triad traits in the allegation-victim condition and David perceived as higher on Dark Triad traits in the assault-victim condition—Emma: *F*(1, 399) = 7.63, *p* = .006, *η*_*p*_*²* = .019; David: *F*(1, 399) = 8.05, *p* = .005, *η*_*p*_*²* = .02. There was also a main effect of citer status on Dark Triad scores for David, as citers perceived him as higher on Dark Triad traits across framing conditions, *F*(1, 399) = 5.3, *p* = .022, *η*_*p*_*²* = .013. There was no main effect of citer status on Dark Triad scores for Emma, though; *F*(1, 399) = 1.72, *p* = .19, *η*_*p*_*²* < .01. However, there was a significant interaction between frame and citer status on Dark Triad scores for Emma, *F*(1, 399) = 6.67, *p* = .01, *η*_*p*_*²* = .016. Planned contrasts revealed that citers perceived Emma as lower on Dark Triad traits in the assault-victim condition, where she was framed as the victim, than in the allegation-victim condition, where David was framed as the victim, *t*(399) = 3.35, *p* < .001, *d* = 0.55. For nonciters, on the other hand, there was no difference in Emma’s Dark Triad scores in the assault-victim and allegation-victim conditions, *t*(399) = 0.15, *p* = .88, *d* = 0.02 (Fig 1c). There was no significant interaction between frame and citer status on Dark Triad scores for David, *F*(1, 399) = 1.27, *p* = .26, *η*_*p*_*²* < .01. However, exploratory contrasts revealed that citers rated David higher on Dark Triad traits in the assault-victim condition than in the allegation-victim condition, *t*(399) = 2.49, *p* = .013, *d* = 0.41. In contrast, nonciters perceived David as having similar Dark Triad traits in the assault-victim and allegation-victim conditions, *t*(399) = 1.42, *p* = .16, *d* = 0.18 (Fig 1d).

#### Experiment 1 discussion.

Taken together, these results suggest that victim framing operates similarly whether the label comes from a third party or from the alleged perpetrator. We also found parallel shifts in perceived Dark Triad traits, implying that victim framing shapes trait attributions in addition to general support. Critically, we replicated the finding that the effects of victim framing are most pronounced amongst those who explicitly cite the framing language as impacting their evaluations of the individuals involved in the alleged incident. This pattern is consistent with the social-pragmatic account of victim framing whereby some people treat the victim label as communicating relevant information and incorporate this information into their evaluations while others do not. The absence of any self-vs.-other differences suggests that “self-victim” claims are not discounted as mere self-serving rhetoric when observers treat the victim label as informative. This finding is broadly consistent with “Truth-Default Theory,” which suggests that people tend to assume that other people are being honest unless they have a specific reason to suspect deception [21].

### Experiment 2

In this experiment, we tested whether victim framing effects would extend to a counter-stereotypical situation where a man has accused a woman of sexual assault. See Table 9 for means and SEs.

**Table 9:** Mean support scores by frame, detail, and citer status in Experiment 2.

Detail	Protagonist	Frame		
		*Assault Victim*	*Allegation Victim*	*Baseline*
Sparse	Accuser (David)	4.03 (0.12)	3.71 (0.13)	3.73 (0.12)
	Alleged Perpetrator (Emma)	2.46 (0.12)	2.99 (0.14)	3.06 (0.11)
Rich	Accuser (David)	3.73 (0.16)	3.45 (0.13)	3.76 (0.13)
	Alleged Perpetrator (Emma)	2.62 (0.13)	2.88 (0.13)	2.75 (0.13)
*Total*	*Accuser (David)*	3.88 (0.10)	3.60 (0.10)	3.75 (0.09)
	*Alleged Perpetrator (Emma)*	2.54 (0.09)	2.93 (0.09)	2.90 (0.10)
**Citer Status**	**Protagonist**	**Frame**		
		*Assault Victim*	*Allegation Victim*	**Contrast**
Citer	Accuser (David)	4.26 (0.12)	3.56 (0.15)	***
	Alleged Perpetrator (Emma)	2.31 (0.12)	3.08 (0.15)	***
Nonciter	Accuser (David)	3.51 (0.16)	3.62 (0.13)	ns
	Alleged Perpetrator (Emma)	2.77 (0.13)	2.84 (0.12)	ns

**Note:** Standard errors in parentheses; citer status data collapsed across detail conditions; planned contrasts: ***p < .001; **p < .01; *p < .05

### Overall framing effects

We conducted separate 3 (frame: assault victim vs. allegation victim vs. baseline) × 2 (detail: sparse vs. rich) ANOVAs on *support* scores for both David (the accuser) and Emma (the alleged perpetrator). There was no main effect of detail nor any significant interaction between detail and frame in either analysis; all *F*s < 2.3, all *p*s > .1. However, there was a significant main effect of frame on support for Emma, *F*(2, 596) = 6.26, *p* = .002, *η*_*p*_^*2*^ = .021, though not for David: *F*(2, 596) = 2.24, *p* = .11, *η*_*p*_^*2*^ < .01. This nonsignificant effect on support for David, the male accuser, could reflect cultural resistance to recognizing men as victims of sexual assault [25], reduced statistical power for detecting accuser effects in this counter-stereotypical scenario, or both. Bonferroni-corrected post-hoc tests revealed that participants expressed less support for Emma in the assault-victim condition than in both the allegation-victim and baseline conditions; *t*(596) = 3.17, *p* = .005, *d* = 0.31, and *t*(596) = 2.93, *p* = .011, *d* = 0.29, respectively. Support for Emma did not differ significantly between the allegation-victim and baseline conditions, *t*(596) = 0.22, *p* > .99, *d* = 0.02.

#### Citers versus nonciters.

**Fig 2:**
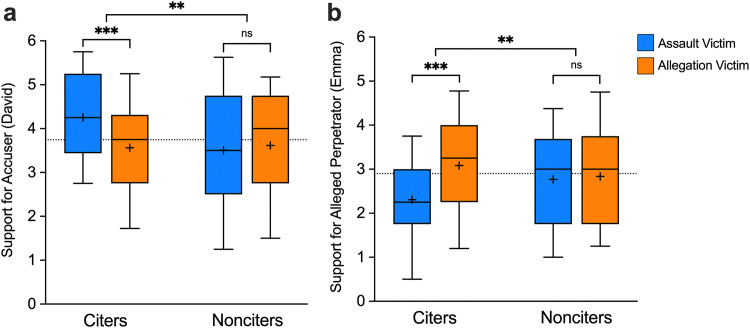
Support for (a) the accuser and (b) the alleged perpetrator in the assault-victim and allegation-victim conditions of Experiment 2, plotted separately for citers and nonciters. Data are collapsed across detail conditions (sparse/rich). Boxes denote the interquartile range, the horizontal middle line indicates the median, whiskers extend from the 10th to the 90th percentiles, and the plus sign denotes the mean. The dashed line indicates the mean in the baseline condition. ***p < .001; **p < .01; *p < .05. Citers showed a robust framing effect on support for both the accuser and alleged perpetrator, while nonciters expressed similar levels of support regardless of framing condition.

Across the two victim framing conditions, 44.3% of participants were classified as citers. Since there was no effect of detail in the previous analyses, we removed this factor and conducted separate 2 (frame: assault victim vs. allegation victim) × 2 (citer status: citer vs. nonciter) ANOVAs on support for each protagonist to assess any differences between citers and nonciters. There was a significant main effect of frame in both analyses—support for David, the accuser: *F*(1, 402) = 4.45, *p* = .036, *η*_*p*_^*2*^ = .011; support for Emma, the alleged perpetrator: *F*(1, 402) = 10.84, *p* = .001, *η*_*p*_^*2*^ = .026. There was also a main effect of citer status on support for David, *F*(1, 402) = 6.26, *p* = .013, *η*_*p*_^*2*^ = .015, but not on support for Emma, *F*(1, 402) = 0.71, *p* = .40, *η*_*p*_^*2*^ < .01. These main effects were qualified by a significant interaction between frame and citer status in both analyses—support for David: *F*(1, 402) = 8.32, *p* = .004, *η*_*p*_^*2*^ = .02; support for Emma: *F*(1, 402) = 7.68, *p* = .006, *η*_*p*_^*2*^ = .019; Planned contrasts revealed that the overall framing effect was driven by citers: for them, support for David was higher in the assault-victim condition than the allegation-victim condition, *t*(402) = 3.34, *p* < .001, *d* = 0.50, whereas support for Emma was higher in the allegation-victim condition than the assault-victim condition, *t*(402) = 4.05, *p* < .001, *d* = 0.61. For nonciters, support for David was similar in the allegation-victim and assault-victim conditions, *t*(402) = 0.58, *p* = .56, *d* = 0.08, as was support for Emma, *t*(402) = 0.39, *p* = .7, *d* = 0.05 (Fig 2).

#### Experiment 2 discussion.

Taken together, these results suggest that explicit victim framing operates similarly in counter-stereotypical sexual assault cases (woman assaults man) as in stereotypical cases (man assaults woman), though the overall effects were somewhat attenuated in the counter-stereotypical scenario. This attenuation is consistent with research showing that there may be some cultural resistance to viewing men as victims of sexual assault [24,25,38]. As in prior research on victim framing [2], the rich vs. sparse detail manipulation did not affect the pattern of results, implying that it is the victim label, rather than narrative elaboration about the alleged incident, that drives the effect when it occurs. Consistent with Experiment 1 and past research [2], victim framing effects were concentrated among citers: those who explicitly relied on the victim language in forming their evaluations showed the familiar shift in support, while nonciters did not. That pattern continues to align with a social-pragmatic account of victim framing.

### Experiment 3

In this experiment, we tested whether victim framing would be effective in situations involving same-sex allegations of sexual assault. See Table 10 for means and SEs.

**Table 10:** Mean support scores by frame, character gender, and citer status in Experiment 3.

Measure	Gender	Protagonist	Frame		
			*Assault Victim*	*Allegation Victim*	*Baseline*
Support	Men	Accuser	4.81 (0.11)	4.33 (0.13)	4.38 (0.11)
		Alleged Perpetrator	2.14 (0.14)	2.43 (0.14)	2.70 (0.11)
	Women	Accuser	4.29 (0.12)	4.20 (0.12)	4.30 (0.12)
		Alleged Perpetrator	2.73 (0.12)	2.94 (0.13)	2.68 (0.12)
	*Total*	*Accuser*	4.55 (0.08)	4.27 (0.09)	4.34 (0.08)
		*Alleged Perpetrator*	2.44 (0.09)	2.68 (0.10)	2.69 (0.08)
**Citer Status**	**Gender**	**Protagonist**	**Frame**		
			*Assault Victim*	*Allegation Victim*	**Contrast**
Citer	Men	Accuser	4.87 (0.20)	4.09 (0.23)	
	Alleged Perpetrator	Alleged Perpetrator	2.14 (0.24)	2.69 (0.24)	
	Women	Accuser	4.56 (0.19)	4.16 (0.19)	
	Alleged Perpetrator	Alleged Perpetrator	2.12 (0.20)	2.91 (0.19)	
	*Total*	*Accuser*	4.72 (0.14)	4.13 (0.15)	**
		*Alleged Perpetrator*	2.13 (0.16)	2.80 (0.15)	**
Nonciter	Men	Accuser	4.77 (0.13)	4.43 (0.15)	
		Alleged Perpetrator	2.13 (0.17)	2.32 (0.16)	
	Women	Accuser	4.15 (0.15)	4.22 (0.15)	
		Alleged Perpetrator	3.06 (0.13)	2.95 (0.17)	
	*Total*	*Accuser*	4.44 (0.11)	4.33 (0.11)	ns
		*Alleged Perpetrator*	2.63 (0.12)	2.63 (0.12)	ns

**Note:** Standard errors in parentheses; planned contrasts: ***p < .001; **p < .01; *p < .05

### Overall framing effects

First, we conducted two 3 (frame: assault victim vs. allegation victim vs. baseline) × 2 (gender: men vs. women) between-subjects ANOVAs on *support* scores for the two characters in the report: the accuser and the alleged perpetrator.

There were main effects of frame, *F*(2, 552) = 3.22, *p* = .041, *η*_*p*_^*2*^ = .012, and of gender, *F*(2, 552) = 6.27, *p* = .013, *η*_*p*_^*2*^ = .011, on support for the accuser. However, there was no interaction between frame and gender, *F*(2, 552) = 2.21, *p* = .11, *η*_*p*_^*2*^ < .01. Bonferroni-corrected post-hoc tests revealed that participants expressed more support for accusers in the assault-victim condition than in the allegation-victim condition, *t*(552) = 2.43, *p* = .047, *d* = .25, though neither condition differed significantly from the baseline condition (*t*s < 1.8, *p*s > .2). Participants also expressed more support for male accusers than female accusers overall, *t*(552) = 2.5, *p* = .013, *d* = 0.21.

There was no main effect of frame on support for the alleged perpetrator, *F*(2, 552) = 2.77, *p* = .064, *η*_*p*_^*2*^ = .01, but there was a significant main effect of gender, *F*(1, 552) = 12.29, *p* < .001, *η*_*p*_^*2*^ = .022. Overall, participants expressed more support for female alleged perpetrators than male alleged perpetrators, *t*(552) = 3.51, *p* < .001, *d* = 0.3. There was also a significant interaction between frame and gender, *F*(2, 552) = 3.38, *p* = .035, *η*_*p*_^*2*^ = .012. Planned contrasts revealed that, in the scenario involving two women, there were no significant differences in support for the alleged perpetrator across framing conditions (all *t*s < 1.4, all *p*s > .16). In the scenario involving two men, on the other hand, participants expressed more support for the alleged perpetrator in the baseline condition than in the assault-victim condition, *t*(552) = 3.17, *p* = .002, *d* = 0.46. Support for the alleged perpetrator did not significantly differ between the allegation-victim and assault-victim conditions (*p* = .34) or between the allegation-victim and baseline conditions (*p* = .098).

#### Citers versus nonciters.

Across the two victim framing conditions, 34.3% of participants were classified as citers. To assess any differences between citers and nonciters, we conducted two 2 (frame: assault victim vs. allegation victim) × 2 (gender: men vs. women) × 2 (citer status: citer vs. nonciter) between-subjects ANOVAs on support scores for the two characters in the report.

**Fig 3:**
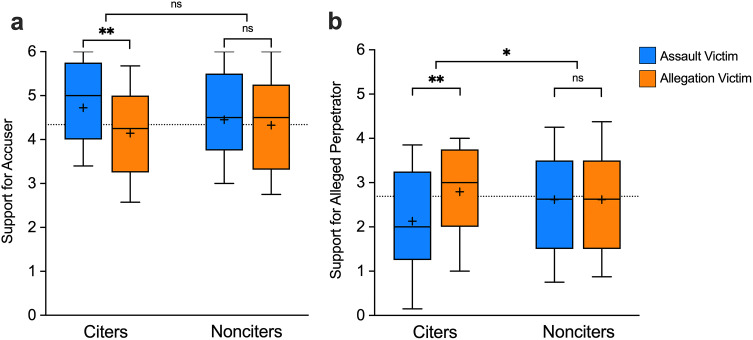
Support for (a) the accuser and (b) the alleged perpetrator in the assault-victim and allegation-victim conditions of Experiment 3, plotted separately for citers and nonciters. Data are collapsed across gender conditions. Boxes denote the interquartile range, the horizontal middle line indicates the median, whiskers extend from the 10th to the 90th percentiles, and the plus sign denotes the mean. The dashed line indicates the mean in the baseline condition. ***p < .001; **p < .01; *p < .05. Citers showed a robust framing effect on support for both the accuser and alleged perpetrator, while nonciters expressed similar levels of support regardless of framing condition.

As in our previous analysis, there were significant main effects of frame and gender on support for the accuser. Overall, participants expressed greater support for the accuser when they were framed as the victim (assault-victim condition) than when the alleged perpetrator was framed as the victim (allegation-victim condition), *F*(1, 365) = 7.98, *p* = .005, *η*_*p*_^*2*^ = .021. Participants also expressed greater support for male accusers than female accusers, *F*(1, 365) = 4.5, *p* = .035, *η*_*p*_^*2*^ = .012. There was no main effect of citer status, nor were there interactions between frame and gender or between gender and citer status, nor a three-way interaction (all *F*s < 2.5, all *p*s > .1). Although the interaction between frame and citer status was not statistically significant, *F*(1, 365) = 3.12, *p* = .078, *η*_*p*_^*2*^ = .008, exploratory contrasts revealed the expected pattern of results: citers expressed greater support for the accuser in the assault-victim condition than in the allegation-victim condition, *t*(365) = 2.82, *p* = .005, *d* = 0.51, while nonciters did no*t* show this effect, *t*(365) = 0.91, *p* = .36, *d* = 0.12 (Fig 3a).

There were significant main effects of frame and gender on support for the alleged perpetrator, mirroring the effects on support for the accuser. Overall, participants expressed greater support for the alleged perpetrator when they were framed as the victim (allegation-victim condition) than when the accuser was framed as the victim (assault-victim condition), *F*(1, 365) = 6.55, *p* = .011, *η*_*p*_^*2*^ = .018. Participants also expressed greater support for female perpetrators than male perpetrators, *F*(1, 365) = 10.04, *p* = .002, *η*_*p*_^*2*^ = .027. There was no main effect of citer status, nor was there an interaction between frame and gender, nor a three-way interaction (all *F*s < 1.2, all *p*s > .28). However, there were significant two-way interactions between frame and citer status, *F*(1, 365) = 5.31, *p* = .022, *η*_*p*_^*2*^ = .014, and between gender and citer status, *F*(1, 365) = 5.94, *p* = .015, *η*_*p*_^*2*^ = .016. Planned contrasts revealed that citers showed the expected framing effect, expressing significantly more support for the alleged perpetrator in the allegation-victim condition than in the assault-victim condition, *t*(365) = 2.99, *p* = .003, *d* = 0.54. Nonciters did not show this effect, *t*(365) = 0.22, *p* = .83, *d* = 0.03 (Fig 3b). Interestingly, citers expressed similar support for the alleged perpetrator regardless of gender, *t*(365) = 0.45, *p* = .65, *d* = 0.08, while nonciters expressed significantly greater support for female alleged perpetrators than male alleged perpetrators, *t*(365) = 4.83, *p* < .001, *d* = 0.62.

#### Experiment 3 discussion.

The effects of explicit victim framing generalized to situations concerning same-sex assault allegations, a context that receives less media attention and for which people likely have weaker cultural scripts than heterosexual assault [39]. Interestingly, the effects were stronger in cases involving two men than two women. This may reflect a mapping from heteronormative scripts of sexual assault that situate male genital penetration as more agentic and “serious” than assaults perpetrated by women [38], or broader patterns of gender-based “moral typecasting” in which men are more readily cast as moral agents and women as patients [24]. In light of these factors, the attenuation of victim framing effects in cases involving two women may be due to contextual resistance to the framing manipulation. However, it could also be an artifact of reduced statistical power within the gender subconditions or simply a statistical anomaly. Critically, however, among citers the expected framing effect was observed regardless of the genders of the individuals, consistent with the social-pragmatic account of victim framing.

### Experiment 4

In this experiment, we examined (a) whether victim framing effects would extend to an alleged case of physical domestic abuse, and (b) whether victim framing effects are comparable for cases involving familiar celebrities and strangers. See Table 11 for means and SEs.

**Table 11:** Mean support scores by frame, celebrity status, and citer status in Experiment 4.

Measure	Celebrity Status	Protagonist	Frame		
			*Assault Victim*	*Allegation Victim*	*Baseline*
Support	Celebrity	Accuser	4.22 (0.12)	3.78 (0.13)	3.75 (0.11)
		Alleged Perpetrator	2.38 (0.12)	2.97 (0.13)	2.93 (0.10)
	Stranger	Accuser	4.67 (0.10)	3.90 (0.13)	4.21 (0.11)
		Alleged Perpetrator	2.07 (0.13)	2.76 (0.12)	2.43 (0.11)
	*Total*	*Accuser*	4.44 (0.08)	3.84 (0.09)	3.98 (0.08)
		*Alleged Perpetrator*	2.23 (0.09)	2.87 (0.09)	2.67 (0.08)
**Citer Status**	**Celebrity Status**	**Protagonist**	**Frame**		
			*Assault Victim*	*Allegation Victim*	**Contrast**
Citer	Celebrity	Accuser	4.76 (0.21)	3.53 (0.23)	
		Alleged Perpetrator	2.03 (0.22)	3.49 (0.20)	
	Stranger	Accuser	4.76 (0.13)	3.17 (0.20)	
		Alleged Perpetrator	1.85 (0.18)	3.42 (0.18)	
	*Total*	*Accuser*	4.76 (0.12)	3.36 (0.16)	***
		*Alleged Perpetrator*	1.93 (0.14)	3.46 (0.14)	***
Nonciter	Celebrity	Accuser	2.93 (0.14)	3.93 (0.14)	
		Alleged Perpetrator	2.56 (0.14)	2.65 (0.16)	
	Stranger	Accuser	4.59 (0.14)	4.29 (0.14)	
		Alleged Perpetrator	2.24 (0.17)	2.41 (0.15)	
	*Total*	*Accuser*	4.23 (0.11)	4.11 (0.10)	ns
		*Alleged Perpetrator*	2.42 (0.11)	2.53 (0.11)	ns

**Note:** Standard errors in parentheses; planned contrasts: ***p < .001; **p < .01; *p < .05

#### Overall framing effects.

First, we conducted two 3 (frame: assault victim vs. allegation victim vs. baseline) × 2 (celebrity status: celebrity vs. stranger) between-subjects ANOVAs on *support* scores for the two characters in the report: (Vicky/Sarah) Park, the accuser, and Nick (Cage/Hatch), the alleged perpetrator. There was a main effect of frame on support for both protagonists—Park: *F*(2, 571) = 14.46, *p* < .001, *η*_*p*_*²* = .048; Nick: *F*(2, 571) = 15.24, *p* < .001, *η*_*p*_*²* = .051. Bonferroni-corrected post-hoc tests revealed that support for Park was significantly higher in the assault-victim condition, where she was framed as the victim, than in both the allegation-victim and baseline conditions; *t*(571) = 5.14, *p* < .001, *d* = 0.52, and *t*(594) = 3.91, *p* < .001, *d* = 0.4, respectively. The difference in support for Park between the allegation-victim and baseline conditions was not statistically significant, *t*(571) = 1.19, *p* = .71, *d* = 0.12. In a parallel fashion, support for Nick was significantly lower in the assault-victim condition than in both the allegation-victim and baseline conditions; *t*(571) = 5.37, *p* < .001, *d* = 0.54, and *t*(594) = 3.77, *p* < .001, *d* = 0.39, respectively. The difference in support for Nick between the allegation-victim and baseline conditions was not statistically significant, *t*(571) = 1.54, *p* = .37, *d* = 0.16.

There was also a significant main effect of celebrity status on support for both protagonists—Park: *F*(1, 571) = 12.63, *p* < .001, *η*_*p*_*²* = .022; Nick: *F*(1, 571) = 11.88, *p* < .001, *η*_*p*_*²* = .020. Overall, participants supported Park more when she accused a stranger (Hatch) of assault than when she accused a celebrity (Cage), and supported Nick more when he was a celebrity than when he was a stranger. This suggests that people may be more likely to question the motivations of women who accuse famous men of assault, perhaps suspecting an ulterior motive (e.g., attention, money) [31,32,40]. Critically, however, there was no interaction between frame and celebrity status on support for either protagonist—Park: *F*(2, 571) = 1.24, *p* = .29, *η*_*p*_*²* < .01; Nick: *F*(2, 571) = 0.75, *p* = .47, *η*_*p*_*²* < .01. This suggests victim framing effects are comparable in cases involving celebrities and strangers.

#### Citers versus nonciters.

**Fig 4:**
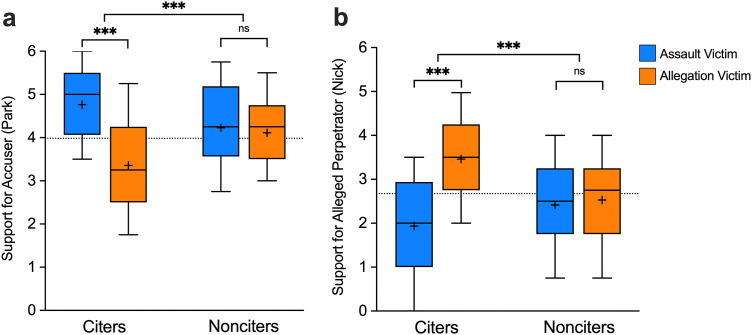
Support for (a) the accuser and (b) the alleged perpetrator in the assault-victim and allegation-victim conditions of Experiment 4, plotted separately for citers and nonciters. Data are collapsed across celebrity status conditions. Boxes denote the interquartile range, the horizontal middle line indicates the median, whiskers extend from the 10th to the 90th percentiles, and the plus sign denotes the mean. The dashed line indicates the mean in the baseline condition. ***p < .001; **p < .01; *p < .05. Citers showed a robust framing effect on support for both the accuser and alleged perpetrator, while nonciters expressed similar levels of support regardless of framing condition.

Across the two victim framing conditions, 37.4% of participants were classified as citers. We conducted two 2 (frame: assault victim vs. allegation victim) × 2 (celebrity status: celebrity vs. stranger) × 2 (citer status: citer vs. nonciter) between-subjects ANOVAs on support scores for the two characters in the report. As in our previous analyses, there was a main effect of frame on support for both protagonists—Park: *F*(1, 382) = 42.89, *p* < .001, *η*_*p*_*²* = .10; Nick: *F*(1, 382) = 43.57, *p* < .001, *η*_*p*_*²* = .10. However, these effects were qualified by significant interactions between frame and citer status—support for Park: *F*(1, 382) = 27.7, *p* < .001, *η*_*p*_*²* = .068; support for Nick: *F*(1, 382) = 30.92, *p* < .001, *η*_*p*_*²* = .075. As in our earlier experiments, planned contrasts revealed that the overall framing effect was driven by citers: for them, support for Park was higher in the assault-victim condition than the allegation-victim condition, *t*(382) = 7.47, *p* < .001, *d* = 1.24, whereas support for Nick was higher in the allegation-victim condition than the assault-victim condition, *t*(382) = 7.69, *p* < .001, *d* = 1.28. However, for nonciters, support for the two protagonists was similar in the allegation-victim and assault-victim conditions —Park: *t*(382) = 1.05, *p* = .29, *d* = 0.14; Nick: *t*(382) = 0.85, *p* = .4, *d* = 0.11; (Fig 4).

None of the other main effects or interactions were statistically significant in these analyses (all *F*s < 3.6, all *p*s > .06), with one exception: a significant citer status × celebrity status interaction on support for Park, the accuser, *F*(1, 382) = 8.36, *p* = .004, *η*_*p*_*²* = .021. Planned contrasts revealed that citers supported Park roughly equally when she accused a celebrity versus a stranger of assault, *t*(382) = 0.96, *p* = .34, *d* = 0.16, but nonciters expressed significantly more support for Park when she accused a stranger than a celebrity, *t*(382) = 3.49, *p* < .001, *d* = 0.45.

#### Experiment 4 discussion.

Taken together, these results suggest that victim framing generalizes to cases involving alleged physical assault and operates in the now-familiar way: framing one party as the victim reliably shifts support, but primarily among participants who explicitly cite that language as influencing their evaluations. We also found a robust main effect of celebrity status: people supported the alleged perpetrator more, and the accuser less, when the accused was a celebrity rather than a stranger. This aligns with prior research showing that fame predicts decreased responsibility attributions and moral condemnation in rape cases [31,32]. Crucially, though, there was no interaction between frame and celebrity status. This is consistent with the social-pragmatic account: citers treated the victim label as informative regardless of notoriety, whereas nonciters discounted the label and instead relied mostly on prior beliefs about motives and credibility. Allegations against celebrities make ulterior motives more salient for accusers and invoke familiarity or halo effects [31,32], which appears to have selectively depressed support among nonciters, as evidenced by the significant interaction between citer status and celebrity status on accuser support. One potential limitation in this study is that we named a real celebrity, which may have evoked idiosyncratic associations that could affect the generalizability of these findings. However, the core picture is consistent: while celebrity status may shape how people evaluate accusations of domestic violence, victim framing functions as a critical, informative cue, at least for some people.

### Experiment 5

In this experiment, we examined whether victim framing effects would extend to an incident involving police violence, where the act itself—a police officer shooting a civilian—is not under dispute. Instead, only the motivations of the protagonists remain ambiguous. See Table 12 for means and SEs.

**Table 12:** Mean support scores by frame and citer status in Experiment 5.

Measure	Protagonist	Frame		
		*Civilian Victim*	*Police Victim*	*Baseline*
Support	Civilian	3.78 (0.09)	3.24 (0.11)	3.56 (0.10)
	Police Officer	2.61 (0.10)	3.12 (0.12)	2.80 (0.10)
**Citer Status**	**Protagonist**	**Frame**		
		*Civilian Victim*	*Police Victim*	**Contrast**
Citer	Civilian	4.27 (0.16)	2.99 (0.20)	***
	Police Officer	2.28 (0.17)	3.45 (0.23)	***
Nonciter	Civilian	3.57 (0.11)	3.35 (0.13)	ns
	Police Officer	2.75 (0.11)	2.97 (0.14)	ns

**Note:** Standard errors in parentheses; planned contrasts: ***p < .001; **p < .01; *p < .05

#### Overall framing effects.

First, we conducted two one-way ANOVAs on support scores for each protagonist, with frame as the independent variable. There was a main effect of frame on support for both protagonists—Jamal (the civilian): *F*(2, 601) = 7.09, *p* < .001, *η*_*p*_^*2*^ = 0.023; John (the police officer): *F*(2, 601) = 6.07, *p* = .002, *η*_*p*_^*2*^  = .020. Bonferroni-corrected post-hoc tests showed that support for Jamal was significantly higher in the civilian-victim condition, where he was framed as the victim, than in the police-victim condition, *t*(601) = 3.75, *p* < .001, *d* = .37. Support for Jamal did not differ significantly between the civilian-victim and baseline conditions, *t*(601) = 1.55, *p* = .36, *d* = 0.16, nor between the police-victim and baseline conditions, *t*(601) = 2.20, *p* = .085, *d* = 0.22. In a parallel fashion, support for John was significantly higher in the police-victim condition, where he was framed as the victim, than in the civilian-victim condition, *t*(601) = 3.45, *p* = .002, *d* = 0.34. Support for John did not differ significantly between the civilian-victim and baseline conditions, *t*(601) = 1.29, *p* = .59, *d* = 0.13, nor between the police-victim and baseline conditions, *t*(601) = 2.16, *p* = .093, *d* = 0.22.

#### Citers versus nonciters.

**Fig 5:**
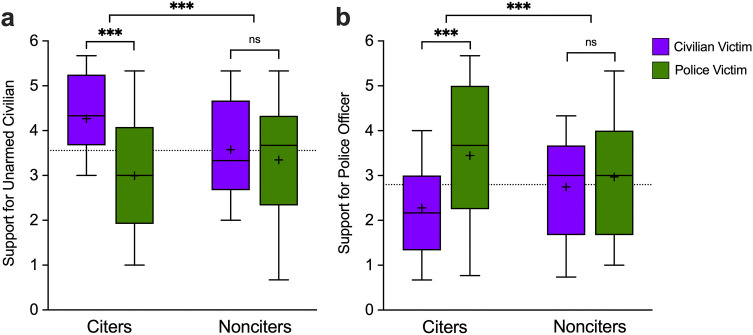
Support for (a) the civilian and (b) the police officer in the civilian-victim and police-victim conditions of Experiment 5, plotted separately for citers and nonciters. Boxes denote the interquartile range, the horizontal middle line indicates the median, whiskers extend from the 10th to the 90th percentiles, and the plus sign denotes the mean. The dashed line indicates the mean in the baseline condition. ***p < .001; **p < .01; *p < .05. Citers showed a robust framing effect on support for both the civilian and police officer, while nonciters expressed similar levels of support regardless of framing condition.

Across the two victim framing conditions, 30.3% of participants were classified as citers. We conducted two 2 (frame: civilian victim vs. police victim) × 2 (citer status: citer vs. nonciter) ANOVAs on support scores for Jamal and John. As in our previous analyses, there was a significant main effect of frame on support for both protagonists—Jamal: *F*(1, 398) = 22.94, *p* < .001, *η*_*p*_^*2*^ = .054; John: *F*(1, 402) = 18.07, *p* < .001, *η*_*p*_^*2*^ = .043—with greater support for Jamal in the civilian-victim condition and greater support for John in the police-victim condition. There was no main effect of citer status in either analysis (*F*s < 1.2, *p*s > .27), but there was a significant interaction between frame and citer status on support for both protagonists—Jamal: *F*(1, 398) = 11.1, *p* < .001, *η*_*p*_^*2*^ = .027; John: *F*(1, 398) = 8.38, *p* < .001, *η*_*p*_^*2*^  = .021. Planned contrasts revealed that the overall framing effects were driven by citers. For them, support for Jamal was greater in the civilian-victim condition than the police-victim condition, *t*(398) = 4.86, *p* < .001, *d* = 0.88. For nonciters, there was no significant difference in support for Jamal between the civilian-victim and police-victim conditions, *t*(398) = 1.33, *p* = .19, *d* = 0.16 (Fig 5a). Similarly, for citers, support for John was higher in the police-victim condition than in the civilian-victim condition, *t*(398) = 4.28, *p* < .001, *d* = 0.78, whereas for nonciters there was no significant difference in support for John between the police-victim and civilian-victim conditions, *t*(398) = 1.23, *p* = .22, *d* = 0.15 (Fig 5b).

#### Experiment 5 discussion.

Victim framing shifted support in a police shooting scenario where the basic facts of the case were not in dispute: the officer shot and maimed the unarmed civilian. Once again, these effects were driven by those who cited the victim-related language as influencing their evaluations, consistent with the social-pragmatic account. As in the previous experiments, citers seemed to follow the pragmatic implications of the victim frame—boosting their support for the labeled victim and lowering their support for the other party—while nonciters were largely unmoved. Given the implicitly racially-charged nature of the scenario, cued by the protagonists’ names, the resistance of nonciters may partially reflect the influence of preexisting attitudes toward policing, which have been shown to impact evaluations of police violence [35]. Taken together, these findings align with prior work showing that attitudes toward police use of force are shaped by both linguistic framing and ideological orientation [36].

## General discussion

Victimhood has been described as “the most powerful force in morality and politics” [41]. Perceptions of victimhood drive moral condemnation, legal and social outcomes, and political conflict. As a result, people accused of wrongdoing are often described as the “real” victim in an effort to mitigate blame and garner support. Past research has shown that explicit “victim framing” can effectively sway public opinion in cases involving disputed allegations of sexual assault [2]. The central empirical contribution of the present set of studies is to demonstrate that the effects of victim framing generalize across a diverse range of scenarios, providing evidence that this linguistic strategy operates in many contexts, not just in prototypical sexual assault cases.

Across five experiments, we observed significant victim framing effects in scenarios where (a) a man accused of sexual assault self-described as the victim, (b) the stereotypical gender roles were reversed such that a woman was accused of sexually assaulting a man, (c) the sexual assault allegations occurred in cases involving two men or two women; (d) a celebrity or stranger was accused of physically assaulting his girlfriend; and (e) a police officer shot and injured an unarmed civilian. There was some variability in the magnitude of these effects and in baseline support for the protagonists across contexts, suggesting that victim framing does not operate identically in all scenarios. Notably, however, participants who explicitly cited the victim-related language as influencing their evaluations showed robust framing effects in all contexts, expressing elevated support for the victim-framed individual. These results were remarkably consistent across experiments, especially when considering the research was conducted over the course of several years, participants were sampled from multiple crowd-sourcing platforms, there were minor methodological variations across experiments, and the scenarios included counter-stereotypical narratives and even an implicitly racially-charged police shooting.

Taken together, these findings are consistent with a social-pragmatic account of victim framing: in every scenario, a sizable proportion of people (~30–45%) appeared to treat the victim label as communicating relevant information about the individuals involved, increasing their support for the victim-framed party. That is, they appeared to infer that the victim label was chosen for a reason—to indicate who deserved their support—and adjust their evaluations accordingly. That being said, the measure of citing victim-related language was a post-hoc, self-report indicator of attention to the framing language rather than a direct measure of pragmatic inference, and several additional mechanisms may also help explain these findings. For example, the victim label may have led to “moral typecasting” [34], activating a moral agent/patient schema that constrained subsequent evaluations. As moral patients, victims are generally viewed as less capable of inflicting moral harm. The victim label could also function as a heuristic cue that shortcuts more effortful evaluations of the protagonists given its general cultural connotations. And confirmation bias could selectively direct attention to evidence in the narrative consistent with the labeled individual’s victimhood. These explanations are not mutually exclusive with the social-pragmatic account. In fact, pragmatic inference might serve as the initial mechanism that activates one or more downstream processes, though future research is needed to distinguish among possible mechanistic pathways.

What about those who did not cite the victim language as influencing their evaluations and showed little evidence of victim framing effects (~55–70% of people)? Our exploratory analyses (detailed in the S1 Text) help contextualize this finding. Although there were few characteristics that reliably distinguished citers from nonciters (see Table D in S1 Text), across all experiments a range of demographic and individual difference variables predicted support for the protagonists in expected ways (see Tables A and B in S1 Text). For example, younger, more liberal participants who accept fewer rape myths expressed more support for accusers in sexual assault cases than older, more conservative people who accept more rape myths; people who hold more negative views of sexual minorities expressed less support for accusers in same-sex assault cases; and conservatives supported police officers involved in a shooting more than liberals [2,35,42,43]. Critically, however, victim framing effects—and the interaction between frame and citer status—remained significant in nearly every case when controlling for all of these covariates (and others), suggesting that the impact of victim framing operates at least partially independently of preexisting attitudes (see Table C in S1 Text).

This pattern hints at what underlies “susceptibility” to victim framing. For citers, the victim label appears to function as a trusted informational or heuristic cue that updates their beliefs about who deserves support, even when it contradicts prior expectations or attitudes. For nonciters, on the other hand, the label is either unnoticed, distrusted, or simply overwhelmed by stronger weighting of other narrative details or background beliefs. Motivated reasoning likely plays a role: people whose prior beliefs strongly favor one party (e.g., a Black civilian shot by a White police officer) may be motivated to discount linguistic cues that threaten those commitments. It is also possible that some people simply do not pick up on the pragmatic implications of the victim framing language and are therefore unaffected by its use in the report. Recent research has shown that there is substantial variability in pragmatic sensitivity to certain phrases and grammatical constructions, and that this sensitivity predicts associated framing effects [44]. Therefore, it is likely that some people do not draw the inference that one individual (and not another) is deserving of support just because that individual is the only one tagged with the victim label in a brief news report.

**Fig 6:**
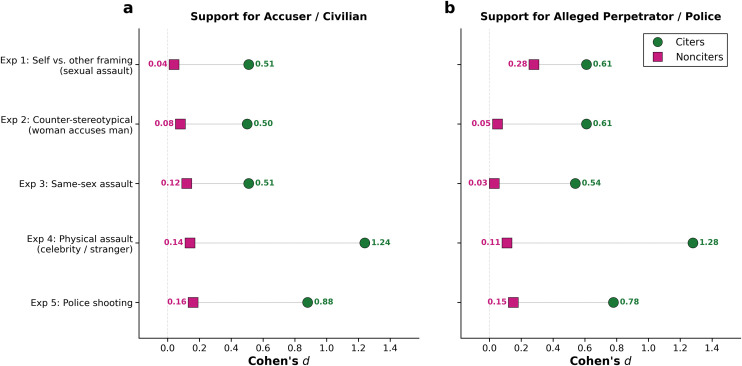
Summary of victim framing effects across all five experiments, computed separately for participants who cited victim-related language as influential (citers) and those who did not (nonciters). Cohen’s *d* reflects the contrast between the two active framing conditions (assault victim vs. allegation victim in Experiments 1-4; civilian victim vs. police victim in Experiment 5). Panel (a) shows effects on support for the accuser/civilian when they were framed as the victim; panel (b) shows effects on support for the alleged perpetrator/police officer when they were framed as the victim. Larger values indicate stronger framing effects in the expected direction (i.e., more support for the victim-framed individual). Across all five experiments, citers showed robust and consistent framing effects—though effect sizes were variable—while nonciters showed minimal differentiation between framing conditions.

The fact that victim framing still swayed ~35% of participants underscores its potency, but the resistance from the remaining ~65% is a reminder that the effects of linguistic framing tend to be modest [11]. Indeed, across all of our experiments, people strongly supported the accusers (and unarmed civilian) over the alleged perpetrators on average, regardless of framing. And among citers, effect sizes for victim framing were mostly in the moderate range (Cohen’s *ds* between 0.50 and 0.88), consistent with the broader linguistic framing literature [11]. See Fig 6. The exception was Experiment 4, the celebrity domestic violence case, where effect sizes were notably larger (Cohen’s *d* = 1.24 and 1.28). This may reflect the relative sparseness of factual details in the Experiment 4 vignette, which gave the victim label more room to influence evaluations in the absence of real competing information. That said, even modest effects can compound when framing language is repeated across millions of media exposures, potentially shaping public opinion about even hotly debated topics.

### Limitations

The present set of studies has several limitations that future research should address. First, our participants were only exposed to victim framing in a brief news report concerning fictionalized cases of alleged wrongdoing. In contrast, real-world judgments about moral disputes are shaped by dozens of factors not captured here, including the credibility and reputation of news sources, cumulative media exposure to a story over time, competing narratives from different parties, visual evidence and emotional testimony (e.g., courtroom footage, social media posts), social identity cues and the opinions of friends and community members, and the broader historical and cultural context in which the allegations occur. Whether victim framing effects persist, accumulate, or wash out under these ecological conditions remains an open question.

Relatedly, we only tested the effects of *explicit* victim framing, where the word “victim” was directly applied to a specific individual. This is distinct from general narrative strategies that position someone as a victim through selective emphasis, tone, or other subtle elements of a story without ever using the label itself. We focused on the explicit case because it is a common and recognizable form of the victim framing strategy [2] and is more easily investigated using established methods in the linguistic framing literature [11]. Although subtler forms of victim framing may also shape moral evaluations, this remains to be tested empirically. It is possible that using the victim label explicitly led our participants to become more attuned to victim framing than they would be in real-world media environments.

Second, our participants made judgments without consequences: no real people were affected by their evaluations, and they faced no social accountability for their responses. Social judgments rendered in low-stakes, anonymous contexts may differ from those made publicly or with tangible outcomes on the line.

Third, our citer/nonciter classification, while revealing, remains somewhat crude. We coded whether participants explicitly pasted or mentioned victim-related language as influencing their evaluations, but this may obscure additional heterogeneity in how they processed the reports, and represents only an indirect measure of pragmatic inference. Future work is needed to assess interpretations of the framing language with more granularity.

Fourth, we did not manipulate certain factors that are likely to moderate the impact of victim framing under the social-pragmatic account, such as source credibility. We might predict, for example, that liberal participants would be less impacted by victim framing when reading a *Fox News* story than a *New York Times* story—and vice versa for conservatives—because they do not trust the author’s partisan leanings.

Finally, our sample, while relatively large and reasonably diverse, leaned more liberal and educated than the general U.S. population, and we did not examine any cross-cultural variation. This limits the generalizability of the findings. Moral judgments related to victimhood are likely shaped by cultural narratives about vulnerability, responsibility, and justice that may differ substantially across societies. People from cultures with different conceptions of honor, masculinity, or gender roles, for example, might respond differently to scenarios where specific individuals are cast as victims (e.g., men who claim to be victims of assault). Whether victim framing effects generalize to other cultural contexts therefore remains to be seen.

### Conclusions and future directions

Despite its limitations, the present research has important implications for understanding the relationships among linguistic framing, social cognition, and moral reasoning. Victimhood has become central to political and moral debate, with competing groups claiming victim status to elevate their grievances and delegitimize their opponents [41]. Our results show that explicit victim framing is more than a rhetorical flourish: across five diverse scenarios, the victim label genuinely shaped how a substantial proportion of people evaluated moral disputes, even when controlling for preexisting attitudes that strongly predicted evaluations on their own.

These findings sit at the intersection of three broader themes. First, they add to a growing literature on the power of basic lexical choices to influence attitudes and decision-making [11]. Second, they align with the view that pragmatic inference plays a key role in how people interpret language and, consequently, how subtle linguistic choices shape social judgments [2,9–11,44]. And third, they carry practical stakes: understanding when, how, and why labeling someone a victim shapes observer reactions is directly relevant to journalists, advocates, and policymakers who discuss morally charged issues. That said, real-world communication about allegations of wrongdoing typically includes narrative repetition, visual and social media, source credibility information, and contextual factors that our studies could not capture. Therefore, our findings should be understood as demonstrating the potential influence of one specific linguistic strategy within a broader communicative ecosystem.

Looking ahead, several lines of future research would strengthen and extend the present findings. First, directly manipulating pragmatic cues such as source credibility would help test whether the effects of victim framing track predictions of the social-pragmatic account specifically. Relatedly, future work could assess individual differences in pragmatic sensitivity to victim labeling and examine whether such sensitivity predicts the magnitude of victim framing effects, as in recent work on grammatical framing [44]. This would provide a more direct test of the role of pragmatic inference in victim framing than the post-hoc citer classification used here. Additionally, a longitudinal study design with multiple sessions could test whether the modest victim framing effects we observed accumulate or attenuate with repeated exposure to the framing narrative. Finally, extending the paradigm to other languages and non-U.S. samples would help determine whether victim framing operates similarly in cultural contexts where norms about victimhood, authority, and gender differ. Ongoing work in our labs is beginning to address several of these questions. In the meantime, the present findings serve as a reminder to all who communicate about morally charged issues: language choices matter, even seemingly small ones.

## Supporting information

S1 Text Supplemental analyses and pilot study. Predictors of support for the accuser/civilian (Table A) and alleged perpetrator/police (Table B); effects of victim framing controlling for participant characteristics (Table C); predictors of citer status (Table D); component-level analyses of individual support measures (Table E); and Experiment 5 pilot study methods, results, and discussion with complete stimuli (Table F).
(DOCX)
