# Supplementary material for: Victim framing shapes attitudes across diverse contexts
Source: PLoS One. 2026 Jun 12;21(6):e0351416. doi: 10.1371/journal.pone.0351416 (PMC13262844; doi:10.1371/journal.pone.0351416)
Supplement: S1 Text — Predictors of support for the accuser/civilian (Table A) and alleged perpetrator/police (Table B); effects of victim framing controlling for participant characteristics (Table C); predictors of citer status (Table D); component-level analyses of individual support measures (Table E); and Experiment 5 pilot study methods, results, and discussion with complete stimuli (Table F). (DOCX) [file pone.0351416.s001.docx]

**Supplemental Material (S1 Text)**

Victim framing shapes attitudes across diverse contexts

Stephen J. Flusberg, Asher Donnelly, J. D. Jarolimek, Esmé Nix, Lili B. Davis, Boshang Yin, Lindsey Anderson, Dylan Ciolfi, & Kevin J. Holmes

**Contents:**

1. Predictors of support
2. Effects of victim framing controlling for participant characteristics
3. Predictors of citer status
4. Analysis of the individual components of support
5. Experiment 5: pilot study

**1. Predictors of support**

We conducted a series of exploratory multiple regression analyses to assess which participant characteristics predicted support for the two protagonists in each experiment. Every analysis included participant age, gender (female = 1; male = 0; other genders were excluded), political ideology (1 = very liberal; 5 = very conservative), highest level of education completed (1 = some high school; 9 = doctorate or professional degree), and annual household income (1 = less than $25,000; 7 = more than $200,000) as covariates. We also included the additional measures uniquely associated with each experiment (e.g., social desirability scale [SDS] in Experiment 1; see main manuscript Methods and Table 2). Note that experimental conditions were not included in these analyses, so these findings reflect general support for the protagonists, collapsing across frame and, e.g., self- vs. other-framing (Experiment 1) or celebrity vs. stranger status (Experiment 4).

*Support for the accuser/civilian*. Across all experiments, support for the accuser/civilian was higher among political liberals than conservatives. For cases involving allegations of sexual assault (Experiments 1-3), support was higher among younger than older people. Gender was only a significant predictor in Experiment 4, with women expressing greater support than men for a situation involving allegations of domestic violence. Several of the unique additional measures also predicted support for the accuser in expected ways. Participants who scored higher on a measure of Dark Triad traits (Experiment 1), believed more rape myths (Experiments 2 and 3), held more negative attitudes toward lesbians and gay men (Experiment 3), and expressed greater trust in police legitimacy (Experiment 5) all indicated lower support for the accuser/civilian. Neither annual household income nor highest level of education completed was a significant predictor in any scenario, nor were any of the other additional measures, including, notably, a measure of socially desirable responding (Experiment 1). See Table A for detailed results.

**Table A:** Predictors of support for the accuser (Experiments 1-4) or civilian (Experiment 5). Significant predictors are in bold.

|  | **Experiment 1** | | **Experiment 2** | | **Experiment 3** | | **Experiment 4** | | **Experiment 5** | |
| --- | --- | --- | --- | --- | --- | --- | --- | --- | --- | --- |
| **Model Fit** | *F*(7, 587) = 17.94,  *p* < .001; R^2^ = .176 | | *F*(7, 587) = 25.40,  *p* < .001; R^2^ = .232 | | *F*(8, 538) = 17.23,  *p* < .001; R^2^ = .204 | | *F*(5, 568) = 10.17,  *p* < .001; R^2^ = .082 | | *F*(7, 586) = 53.82,  *p* < .001; R^2^ = .289 | |
| **Predictor** | *β* | *p* | *β* | *p* | *β* | *p* | *β* | *p* | *β* | *p* |
| Age | **-.088** | **.026** | **-.234** | **< .001** | **-.106** | **.008** | .036 | .378 | .027 | .455 |
| Gender (female) | .067 | .084 | .053 | .159 | .070 | .086 | **.178** | **< .001** | -.042 | .233 |
| Political ideology | **-.338** | **< .001** | **-.117** | **.004** | **-.104** | **.031** | **-.210** | **< .001** | **-.260** | **< .001** |
| Education | .037 | .350 | .012 | .753 | -.011 | .799 | .030 | .486 | .044 | .226 |
| Income | .021 | .602 | < -.001 | .996 | -.016 | .708 | -.027 | .540 | .011 | .758 |
| Short Dark Triad Scale (SD3) | **-.205** | **< .001** | - | - | - | - | - | - | - | - |
| Social Desirability Scale (SDS) | -.067 | .083 | - | - | - | - | - | - | - | - |
| Acceptance of Interpersonal Violence (AIV) | - | - | .017 | .720 | -.098 | .064 | - | - | - | - |
| Acceptance of Rape Myths (IRMA) | - | - | **-.337** | **< .001** | **-.220** | **< .001** | - | - | - | - |
| Attitudes Toward Lesbians and Gay Men (ATLG) | - | - | - | - | **.118** | **.021** | - | - | - | - |
| Attitudes Toward the Criminal Legal System Scale (ATCLS) | - | - | - | - | - | - | - | - | .058 | .265 |
| Attitudes Towards Police Legitimacy Scale (ATPLS) | - | - | - | - | - | - | - | - | **-.391** | **< .001** |

*Support for the alleged perpetrator/police*. Across all experiments—except Experiment 2, where the alleged perpetrator of sexual assault was a woman—support for the alleged perpetrator/police was higher among political conservatives than liberals. Older participants supported the alleged perpetrator more in Experiments 2 and 3, and men supported the alleged perpetrator more in Experiments 1 and 4. Support for the alleged perpetrator/police was also greater among those who scored higher on a measure of Dark Triad traits (Experiment 1), believed more rape myths (Experiments 2 and 3), indicated more acceptance of interpersonal violence (Experiment 3, but not 2), and expressed greater trust in police legitimacy (Experiment 5). None of the other measures was a reliable predictor of support for the alleged perpetrator/police. See Table B for detailed results.

**Table B:** Predictors of support for the alleged perpetrator (Experiments 1-4) or police officer (Experiment 5). Significant predictors are in bold.

|  | **Experiment 1** | | **Experiment 2** | | **Experiment 3** | | **Experiment 4** | | **Experiment 5** | |
| --- | --- | --- | --- | --- | --- | --- | --- | --- | --- | --- |
| **Model Fit** | *F*(7, 587) = 12.42,  *p* < .001; R^2^ = .129 | | *F*(7, 587) = 9.54,  *p* < .001; R^2^ = .102 | | *F*(8, 538) = 8.90,  *p* < .001; R^2^ = .117 | | *F*(5, 568) = 8.13,  *p* < .001; R^2^ = .067 | | *F*(7, 586) = 53.82,  *p* < .001; R^2^ = .415 | |
| **Predictor** | ***β*** | ***p*** | ***β*** | ***p*** | ***β*** | ***p*** | ***β*** | ***p*** | ***β*** | ***p*** |
| Age | .061 | .131 | **.239** | **<.001** | **.167** | **< .001** | -.029 | .487 | -.014 | .689 |
| Gender (female) | **-.095** | **.017** | -.009 | .816 | .013 | .767 | **-.175** | **< .001** | .048 | .143 |
| Political ideology | **.298** | **< .001** | .019 | .654 | **.125** | **.013** | **.166** | **< .001** | **.191** | **< .001** |
| Education | -.010 | .811 | -.023 | .587 | .008 | .864 | -.041 | .348 | -.061 | .070 |
| Income | .033 | .417 | .020 | .639 | .039 | .381 | .065 | .144 | .030 | .375 |
| Short Dark Triad Scale (SD3) | **.124** | **.003** | - | - | - | - | - | - | - | - |
| Social Desirability Scale (SDS) | < .001 | .986 | - | - | - | - | - | - | - | - |
| Acceptance of Interpersonal Violence (AIV) | - | - | -.009 | .858 | **.130** | **.019** | - | - | - | - |
| Acceptance of Rape Myths (IRMA) | - | - | **.201** | **< .001** | **.165** | **.008** | - | - | - | - |
| Attitudes Toward Lesbians and Gay Men (ATLG) | - | - | - | - | .019 | .720 | - | - | - | - |
| Attitudes toward the Criminal Legal System Scale (ATCLS) | - | - | - | - | - | - | - | - | -.074 | .120 |
| Attitudes Towards Police Legitimacy Scale (ATPLS) | - | - | - | - | - | - | - | - | **.545** | **< .001** |

**2. Effects of victim framing when controlling for participant characteristics**

Next, we examined whether the victim framing effects we observed—and specifically the frame × citer status interaction—remained significant when controlling for participant characteristics. To address this, we added frame (*assault/civilian victim* vs. *allegation/police victim*; baseline condition excluded) and citer status (*citer* vs. *nonciter*) as factors into the covariate-only models, along with the frame × citer status interaction. See Table C for measures of improved model fit over the covariate-only models, as well as the unstandardized coefficients and *p*-values for the new terms in the models.

**Table C:** Effects of frame and citer status on support for the accuser/civilian and alleged perpetrator/police across experiments, controlling for demographics and individual difference measures. Significant predictors are in bold.

| **Support for the Accuser/Civilian** | | | | | | | | | | |
| --- | --- | --- | --- | --- | --- | --- | --- | --- | --- | --- |
|  | **Experiment 1** | | **Experiment 2** | | **Experiment 3** | | **Experiment 4** | | **Experiment 5** | |
| **Improved model fit** | Δ*F*(3, 390) = 7.57,  *p* < .001; ΔR^2^ = .044 | | Δ*F*(3, 390) = 9.00,  *p* < .001; ΔR^2^ = .049 | | Δ*F*(3, 352) = 6.20,  *p* < .001; ΔR^2^ = .039 | | Δ*F*(3, 379) = 18.47,  *p* < .001; ΔR^2^ = .115 | | Δ*F*(3, 384) = 7.23,  *p* < .001; ΔR^2^ = .039 | |
| **Predictor** | ***B*** | ***p*** | ***B*** | ***p*** | ***B*** | ***p*** | ***B*** | ***p*** | ***B*** | ***p*** |
| Frame (assault/ civilian victim) | .011 | .950 | -.158 | .324 | .208 | .129 | .213 | .138 | **.370** | **.015** |
| Citer status (citer) | .061 | .681 | -.053 | .760 | -.145 | .410 | **-.678** | **< .001** | -.208 | .285 |
| Frame × citer status | **.663** | **.008** | **.856** | **< .001** | **.506** | **.031** | **1.127** | **< .001** | .540 | .054 |
| **Support for the Alleged Perpetrator/Police** | | | | | | | | | | |
|  | **Experiment 1** | | **Experiment 2** | | **Experiment 3** | | **Experiment 4** | | **Experiment 5** | |
| **Improved model fit** | Δ*F*(3, 390) = 8.99,  *p* < .001; ΔR^2^ = .055 | | Δ*F*(3, 390) = 6.08,  *p* < .001; ΔR^2^ = .039 | | Δ*F*(3, 352) = 5.82,  *p* < .001; ΔR^2^ = .043 | | Δ*F*(3, 379) = 20.99,  *p* < .001; ΔR^2^ = .131 | | Δ*F*(3, 384) = 6.62,  *p* < .001; ΔR^2^ = .030 | |
| **Predictor** | ***B*** | ***p*** | ***B*** | ***p*** | ***B*** | ***p*** | ***B*** | ***p*** | ***B*** | ***p*** |
| Frame (assault/ civilian victim) | **-.426** | **.010** | -.065 | .688 | -.036 | .825 | -.198 | .182 | **-.345** | **.017** |
| Citer status (citer) | .153 | .439 | .236 | .179 | .202 | .337 | **.860** | **< .001** | .352 | .056 |
| Frame × citer status | -.527 | .057 | **-.677** | **.006** | **-.797** | **.004** | **-1.268** | **< .001** | -.441 | .097 |

In every case, adding frame, citer status, and their interaction significantly improved model fit over the covariate-only model (all Δ*F*s > 5.8, *p*s < .001; ΔR^2^s between .03-.131). In the models predicting support for the accuser/civilian, the frame × citer status interaction was statistically significant for Experiments 1-4 (*B*s > .50; *p*s *<* .04), and trended in the expected direction in Experiment 5 (*B* = .540; *p =* .054). In the models predicting support for the alleged perpetrator/police, the frame × citer status interaction was statistically significant for Experiments 2-4 (*B*s < -.67; *p*s *<* .006), and trended in the expected direction in Experiment 1 (*B* = -.527; *p =* .057) and Experiment 5 (*B* = -.441; *p =* .097).

**3. Predictors of citer status**

We next asked whether participants who explicitly cited victim-framing language as influencing their evaluations (*citers*, coded 1) differed from those who did not (*nonciters*, coded 0). For each experiment, we fit a logistic regression predicting citer status with the following factors and covariates: framing condition, any study-specific condition/manipulation (e.g., sparse vs. detailed reports in Experiment 2), demographics (age, gender, political ideology, highest level of education, annual household income), individual-difference measures (e.g., SDS, IRMA, or ATCLS, where applicable), and reading time (i.e., the amount of time the participant spent on the screen that displayed the stimulus paragraph). The baseline condition was excluded from these analyses, as were participants who did not identify as male or female. Omnibus measures of model fit indicated that the logistic regression models were only statistically significant for Experiments 1 and 2. Therefore, these findings should be interpreted cautiously. See Table D for results.

**Table D:** Omnibus logistic regression model fit and predictors of citer status (odds ratios with 95% CIs and *p*-values). Significant predictors are in bold.

|  | **Experiment 1** | | **Experiment 2** | | **Experiment 3** | | **Experiment 4** | | **Experiment 5** | |
| --- | --- | --- | --- | --- | --- | --- | --- | --- | --- | --- |
| **Model Fit** | LR χ²(10) = 19.53,  *p* = .034; AUC = .640 | | LR χ²(X) = 67.32,  *p* < .001; AUC = .731 | | LR χ²(10) =17.88,  *p* = .057; AUC = .630 | | LR χ²(8) = 4.31,  *p* = .828; AUC = .553 | | LR χ²(9) = 3.38,  *p* = .947; AUC = .568 | |
| **Predictor** | **OR [CI]** | ***p*** | **OR [CI]** | ***p*** | **OR [CI]** | ***p*** | **OR [CI]** | ***p*** | **OR [CI]** | ***p*** |
| Age | .996 [.979, 1.01] | .695 | 1.01 [.994, 1.03] | .195 | .988 [.969, 1.01] | .209 | .996 [.979, 1.01] | .627 | .999 [.981, 1.02] | .923 |
| Gender (female) | 1.01 [.650, 1.56] | .977 | 1.12 [.711, 1.75] | .635 | .835 [.510, 1.37] | .475 | 1.01 [.668, 1.54] | .951 | 1.32 [.833, 2.09] | .236 |
| Political ideology | 1.01 [.839, 1.22] | .893 | 1.14 [.914, 1.41] | .251 | 1.17 [.922, 1.49] | .196 | 1.05 [.886, 1.26] | .552 | .937 [.750, 1.17] | .563 |
| Education | .960 [.856, 1.08] | .484 | .937 [.832, 1.06] | .281 | .995 [.885, 1.12] | .928 | 1.08 [.963, 1.21] | .191 | 1.01 [.893, 1.13] | .928 |
| Income | .898 [.771, 1.05] | .163 | .905 [.777, 1.06] | .203 | **1.17 [1.00, 1.36]** | **.048** | 1.00 [.871, 1.15] | .972 | .943 [.807, 1.10] | .464 |
| Reading time | 1.00 [.999, 1.00] | .303 | 1.00 [.998, 1.00] | .531 | 1.00 [.996, 1.00] | .971 | 1.00 [.998, 1.01] | .343 | .999 [.997, 1.00] | .689 |
| Frame (assault/ civilian victim) | 1.28 [.841, 1.95] | .250 | **1.58 [1.03, 2.43]** | **.038** | **1.59 [1.00, 2.51]** | **.048** | 1.15 [.760, 1.74] | .508 | .967 [.623, 1.50] | .881 |
| Framer (self) | **1.87 [1.22, 2.86]** | **.004** | - | - | - | - | - | - | - | - |
| Dark Triad (SD3) | 1.02 [.687, 1.52] | .919 | - | - | - | - | - | - | - | - |
| Soc. Des. (SDS) | **.862 [.749, .993]** | **.039** | - | - | - | - | - | - | - | - |
| AIV | - | - | .812 [.496, 1.33] | .407 | 1.72 [.999, 2.95] | .050 | - | - | - | - |
| IRMA | - | - | .887 [.578, 1.36] | .581 | .816 [.520, 1.28] | .374 | - | - | - | - |
| Detail (sparse) | - | - | **4.80 [3.08, 7.48]** | **< .001** | - | - | - | - | - | - |
| Character gender (women) | - | - | - | - | .818 [.523, 1.28] | .497 | - | - | - | - |
| ATLG | - | - | - | - | 1.02 [.804, 1.30] | .856 | - | - | - | - |
| Celebrity status (stranger) | - | - | - | - | - | - | 1.11 [.732, 1.68] | .624 | - | - |
| ATCLS | - | - | - | - | - | - | - | - | 1.01 [.712, 1.43] | .958 |
| ATPLS | - | - | - | - | - | - | - | - | .973 [.766, 1.24] | .823 |

Overall, there were few significant predictors of citer status, and almost no consistent predictors across experiments. In Experiment 1, participants were more likely to cite the victim framing language in the self-framing condition than the other-framing condition. This suggests people were especially likely to cue in on the pragmatic implications of victim framing when the report centered a protagonist’s own characterization of the event. Participants were also slightly more likely to be a citer when they scored *lower* on socially desirable responding. This suggests that the socially desirable response in these studies would be to affirm that the details of the case—and not the victim framing language—are what most influenced your judgments. This result makes sense on the view that people hope to maintain an image of themselves as rationally evaluating the details of a case as opposed to trusting the anecdotal account of an individual or their supporters. It also provides some evidence against a simple demand characteristic account of victim framing, which would predict that socially desirable responding should be associated with exhibiting a stronger victim framing effect (i.e., being a citer). In Experiments 2 and 3, participants were more likely to be a citer in the *assault victim* condition than in the *allegation victim* condition. This could indicate that people are more likely to accept the pragmatic implications of victim framing when framing supports the alleged victim of sexual assault than the alleged perpetrator. However, these effects were small and inconsistent across studies, and therefore could simply reflect statistical noise. Additionally, participants in Experiment 2 were much more likely to be a citer when the report was sparse than rich in detail. However, this is not surprising given that the rich report contained significantly more details about the incident, which participants may have found informative and therefore highlighted as influencing their evaluations. The only other significant predictor was income in Experiment 3, as participants reporting higher annual household income were slightly more likely to be citers. Given the size of this effect and its absence in every other experiment, it is difficult to interpret.

**4. Analysis of the individual components of support**

The composite support scores used throughout the main manuscript and the above analyses averaged across the individual components of *empathy, believability, harm*, and *responsibility* (reverse-coded) in Experiments 1-4, and *empathy, emotional suffering*, and *responsibility* (reverse-coded) in Experiment 5. To assess whether victim framing differentially affected these individual components, we examined the frame × citer status interaction separately for each component and each protagonist in every experiment (baseline condition excluded). Results are presented in Table E.

**Table E:** Frame × citer status interaction

|  |  | **Exp 1** | **Exp 2** | **Exp 3** | **Exp 4** | **Exp 5** |
| --- | --- | --- | --- | --- | --- | --- |
| **Accuser / Civilian** | |  |  |  |  |  |
|  | Empathy | 8.00 ** | 5.38 * | 1.56 | 21.35 *** | 11.27 *** |
|  | Believability | 8.36 ** | 7.45 ** | 4.74 * | 10.14 ** | — |
|  | Harm / Suffering | 6.15 * | 6.04 * | 1.66 | 27.02 *** | 2.68 |
|  | Responsibility (rev) | 0.19 | 4.11 * | 1.97 | 12.01 *** | 10.57 ** |
| **Alleged Perp. / Police** | |  |  |  |  |  |
|  | Empathy | 2.15 | 4.40 * | 8.96 ** | 28.79 *** | 11.87 *** |
|  | Believability | 4.04 * | 4.47 * | 8.77 ** | 0.01 | — |
|  | Harm / Suffering | 0.93 | 4.03 * | 0.72 | 22.73 *** | 1.47 |
|  | Responsibility (rev) | 3.30 | 7.63 ** | 0.97 | 42.48 *** | 6.66 * |
| *Note*: Cells report *F*-values for the frame × citer status interaction. Degrees of freedom: Exp 1 (1, 397); Exp 2 (1, 402); Exp 3 (1, 368); Exp 4 (1, 386); Exp 5 (1, 398). Experiment 5 used *emotional suffering* rather than *harm* and did not include *believability* because the facts of the case were not in dispute. Responsibility is reverse-coded. **p* < .05; ***p* < .01; ****p* < .001. | | | | | | |

Overall, the frame × citer interaction was significant or trending for the majority of component measures, and the effects were consistently in the same direction as the composite measure. In Experiment 2, the interaction was significant for every component for both the accuser and alleged perpetrator, closely mirroring the composite results. In Experiment 4, the interaction was significant for all components except believability of the alleged perpetrator. However, we did observe some variation across components. For example, in Experiment 1, the interaction was significant for empathy, believability, and harm for the accuser, but not responsibility, and was nonsignificant for all components except believability for the alleged perpetrator. In Experiment 3, the interaction was significant only for believability (accuser) and empathy and believability (alleged perpetrator). In Experiment 5, the interaction was significant for empathy and responsibility but not suffering for both protagonists. Taken together, these patterns suggest that the composite score provides a reasonable summary of individual component framing effects, but that explicit victim framing may most reliably affect perceptions of empathy and believability, with more variable impacts on perceived harm/emotional suffering and responsibility.

**5. Experiment 5: pilot study**

This experiment was our first test of whether the effects of victim framing would generalize beyond intimate partner violence to the context of racially-charged police violence against a civilian. As in Experiment 5, participants were randomly assigned to read a fictionalized news report about a police officer shooting an unarmed civilian that framed either the civilian as the victim, the police officer as the victim, or neither individual as a victim (baseline). Additionally, the report either included or did not include a description of qualified immunity (QI), a policy that grants police officers immunity from civil suits at the discretion of government officials or explicit legal precedent. QI makes it more difficult to hold law enforcement accountable for alleged violent incidents, so we wanted to see whether explaining the concept to participants might moderate the impact of victim framing.

**Participants**

In January 2021, we recruited 626 participants through Amazon Mechanical Turk via the CloudResearch participant-sourcing platform. All participants were at least 18 years old and located in the United States. We limited participants to high performers on MTurk (> 95% rating in at least 100 previous tasks); participants who incorrectly answered an initial attention check question were prevented from completing the study (*n* = 25). The final sample (*N* = 601) had the following demographic characteristics: mean age = 43 (*SD* = 14); gender: 59% women, 40% men, and 1% transgender or non-binary; race/ethnicity: 77% White, 10% African American, 5% Asian/Asian American, 5% Latino/Hispanic; political affiliation: 46% Democrat, 23% Republican, 32% Independent.

**Design, materials, and procedure**

The design of this study was similar to the experiments reported in the main manuscript (especially Experiment 5). We used a 3 (frame: *civilian victim* vs. *police victim* vs. *baseline*) × 2 (QI: *described* vs. *not described*) between-subjects design. Participants were randomly assigned to read one of six fictionalized news reports, each of which described an incident where a police officer shot an unarmed civilian. The report framed either the civilian (Jamal Smith) as the victim of police brutality, the police officer (John O’Neil) as the victim of his duty to enforce the law, or neither party as a victim. The names of the protagonists were selected so that participants would be likely to construe the events along stereotypical racial lines, in line with then-recent media coverage of and public outrage toward instances of White officers allegedly using unprovoked violent tactics toward Black civilians. For half of the participants in each framing condition, the end of the report included a description of QI. See Table F for complete stimuli.

**Table F:** News report stimuli used in Experiment 5 Pilot Study

| Report Section | Frame | | |
| --- | --- | --- | --- |
|  | Civilian Victim | Police Victim | Baseline |
| Headline | Livingston Resident  is a Victim of Police  Violence  (**April 10, 2019)** | Livingston Police  Officer is a Victim of  Enforcing the Law  (**April 10, 2019)** | Livingston Police  Officer and Resident  Await Investigation  (**April 10, 2019)** |
| Description of event (identical for all frame conditions) | A violent encounter last night between a Livingston police officer and a civilian ended with two gunshot wounds, a civilian in the hospital, and outrage in the community. At approximately 10:30 p.m. on April 9, Officer John O’Neil responded to reports of a disturbance in a Safeway parking lot in northeast Livingston, a hub for local gang activity. Upon O’Neil’s arrival at the scene, local resident Jamal Smith emerged from a parked black SUV and allegedly ran towards the police car, prompting O’Neil to shoot him twice in the chest and leg. Paramedics rushed Smith to the hospital, where he is in stable condition and is expected to recover as he awaits legal proceedings. An investigation of the incident is underway. In the meantime, Officer O'Neil has been assigned to desk duty, has been stripped of his firearm, and faces calls for his termination from the force. | | |
|  |  |  |  |
|  |  |  |  |
|  |  |  |  |
| Framing section | “Jamal Smith was shot for no reason,” said a Safeway employee who  witnessed the incident. “I saw the whole situation go down, and Jamal’s  assault is yet another example of callous police violence.” De’ja Smith, Jamal’s wife, also defended her husband. “Jamal was just out running errands for our  family. He’s the victim of this altercation. I pray  that the law will bring him justice, not more trauma.” | “Officer O’Neil acted  swiftly for good reason,” said a Safeway employee  who witnessed the incident. “I saw the whole situation go down, and I thank Officer O’Neil for his dedication to keeping our community safe.” Susan O’Neil, the officer’s wife, also defended her husband. “John was doing his rightful duty as a law enforcement professional. He’s the real victim of this  altercation. I pray that the law will bring him justice, not more trauma.” | “It was a tragic scene,” said a Safeway employee who witnessed the incident. “I saw the whole situation go down. Officer O’Neil acted swiftly in his capacity as a law enforcement professional, yet Jamal got shot when he was just out running errands for his family.” Said another witness, “I’m not sure who’s at fault here. I just hope the law will bring justice, not more  trauma.” |
| Qualified  Immunity  description | Investigators are considering the application of a legal principle known as qualified immunity, which protects law enforcement officials in civil suits if their actions, though possibly unlawful, do not violate any legal precedent. If found applicable, qualified immunity would vacate any charges against O’Neil and confirm that he acted reasonably in his duty as a police officer. | | |
| **Note:** Participants received one of the three headlines, followed by the general description of the event, and then by the corresponding framing section. Half then saw the qualified immunity description, for a total of six unique reports. | | | |

After reading the report, participants responded to the same set of dependent measures as in Experiment 5, with one notable addition for those in the QI-described condition. Specifically, all participants expressed their degree of *empathy* toward the protagonists, indicated how much *emotional suffering* they thought these individuals went through, and rated how *responsible* each of them was for the incident (reverse-coded). These were presented in an interleaved fashion, and which character appeared first was counterbalanced. Scores on these three measures were averaged into a composite *support* score (Cronbach’s ɑ = 0.776 for the civilian, Jamal, and 0.748 for the police officer, John). Next, participants were asked “How do you think the safety of the Livingston community has changed as a result of the incident?” They responded using a Likert scale ranging from 0 (much less safe) to 6 (much more safe). They were then asked “In whose favor do you think the investigation will be resolved?” and selected a response from a multiple-choice list: Jamal Smith, John O’Neil, or Not sure (please explain).

On the following screen, participants completed our *citing* measure, where they copy-pasted which part of the report was most influential in their evaluation of the protagonists, and provided any other information that contributed to their evaluations in a second text box. We used the same automated text-coding procedure to determine whether or not participants cited the victim-framing language in the report (i.e., the words “victim” and/or “trauma”) as influencing their evaluations. Participants in the QI-described condition then responded to two true/false statements about QI: (1) “If found applicable, qualified immunity vacates legal charges against police officers”; (2) “If qualified immunity is applied in a civil suit against John O'Neil, he would not be found guilty of assault.” 76.5% of participants got both questions right, 20.2% got one question right, and 3.3% got both wrong. Finally, participants completed the same two additional measures as in Experiment 5—the Attitudes Towards Police Legitimacy Scale (ATPLS) and the Attitudes Toward the Criminal Legal System Scale (ATCLS)—along with our demographics questions.

**Results**

We carried out the same general set of pre-registered analyses as in Experiments 1-5.

*Overall Framing Effects*. We conducted two 3 (frame: civilian victim vs. police victim vs. baseline) × 2 (QI described vs. not described) ANOVAs on support scores for each protagonist. There was a main effect of frame on support for both protagonists—Jamal, the civilian: *F*(2, 595) = 52.32, *p* < .001, *η_p_²* = .15; John, the police officer: *F*(2, 595) = 22.12, *p* < .001, *η_p_²* = .069. Support for Jamal was significantly lower in the police-victim condition (*M* = 3.10, *SE* = .09) than in either the civilian-victim (*M* = 4.30, *SE* = .09) or baseline (*M* = 4.07, *SE* = .09) condition; Bonferroni-corrected *t*(595) = 9.63, *p* < .001, *d* = 0.96, and *t*(595) = 7.78, *p* < .001, *d* = 0.78, respectively. There was no significant difference in support for Jamal between the civilian-victim and baseline conditions, *t*(595) = 1.84, *p* = .20, *d* = 0.19. In a parallel fashion, support for John was significantly higher in the police-victim condition (*M* = 3.44, *SE* = .10) than in either the civilian-victim (*M* = 2.57, *SE* = .09) or baseline (*M* = 3.16, *SE* = .09) condition; Bonferroni-corrected *t*(595) = 6.52, *p* < .001, *d* = 0.65, and *t*(595) = 4.42, *p* < .001, *d* = 0.44, respectively. There was no significant difference in support for John between the civilian-victim and baseline conditions, *t*(595) = 2.07, *p* = .12, *d* = 0.21. There was no main effect of including the QI description, nor any significant interaction between frame and QI description in either analysis (*F*s < 3.5, *p*s > .062).

*Citers vs. Nonciters.* Across the two victim framing conditions, just 13.9% of participants were classified as citers. Since there was no effect of QI description in the previous analyses, we removed this factor and conducted separate 2 (frame: civilian victim vs. police victim) × 2 (citer status: citer vs. nonciter) ANOVAs on support for each protagonist to assess any differences between citers and nonciters. As in our previous analyses, there was a significant main effect of frame on support for both protagonists, with greater support for Jamal (the civilian) in the civilian-victim condition, *F*(1, 398) = 55.09, *p* < .001, *η_p_²* = .122, and greater support for John (the police officer) in the police-victim condition, *F*(1, 398) = 24.77, *p* < .001, *η_p_²* = .059. There was also a main effect of citer status on support for Jamal, with citers (*M* = 4.35, *SE* = .16) expressing greater support than nonciters (*M* = 3.59, *SE* = .08), *F*(1, 398) = 5.58, *p* = .019, *η_p_²* = .014. There was no main effect of citer status on support for John, nor was there a significant interaction between frame and citer status in either analysis (*F*s < 3, *p*s > .08). However, the frame × citer status interaction was in the expected direction (stronger effect for citers), in particular on support for Jamal, *F*(1, 398) = 2.996, *p* = .084, *η_p_²* = .007.

**Discussion**

We found evidence for a basic victim framing effect in this pilot study, with participants expressing significantly more support for whichever protagonist was framed as the victim relative to the other victim-framing condition and, in some cases, relative to the baseline condition as well. This shows that the effects of victim framing generalize to the domain of police violence. Presenting information about qualified immunity had no impact on participants’ evaluations, however. Although citers tended to support the civilian more than the police officer in general, we found no significant interaction between frame and citer status, in contrast to all of the experiments presented in the main manuscript and to previous published research on victim framing. Notably, there were far fewer citers in this pilot study (< 14%) compared to the other experiments (~30-45%). One possible reason for these discrepancies is that the news reports in this pilot study contained emotionally charged language, including many terms other than “victim” that may have attenuated the influence of the “victim” frame itself (e.g., “shot,” “swiftly,” “assault,” “dedication”). The incident itself was also especially violent, with two gunshots, including one to the chest, which may have been deemed excessive by some no matter the frame. That is why we revised our stimuli for Experiment 5, toning down the language and reducing the severity of the incident.
