# Supplementary material for: Victim framing shapes attitudes across diverse contexts
Source: PLoS One. 2026 Jun 12;21(6):e0351416. doi: 10.1371/journal.pone.0351416 (PMC13262844; doi:10.1371/journal.pone.0351416)
Supplement: Attachment — Submitted filename: Response_to_Reviewers.docx [file pone.0351416.s002.docx]

Response to Reviewers

**5. Review Comments to the Author**

**Please use the space provided to explain your answers to the questions above. You may also include additional comments for the author, including concerns about dual publication, research ethics, or publication ethics. (Please upload your review as an attachment if it exceeds 20,000 characters)**

***Reviewer #1: This is an interesting piece on the weight of victim framing in how people understand perpetrators of offences. It follows up on a recent set of studies on victim framing which looked at men accused of sexual assault where respondents who cited victim-related language as influential believed that the victim language was included because that person deserved their support. This study expands this research to look at contexts beyond accusations of men sexually assaulting women to determine whether the linguistic victim framing applies in different contexts. 5 experiments were conducted, each considering a different type of case. A total of 3,057 participants were sampled, with 2,941 analyzed (with reasons for the decrease explained). Each experiment has so much data that there could very well be enough content for a paper on each one, but this article does a good job of highlighting the key features and key findings of the experiment as a whole.* *The introduction is clearly presented and offers useful context for the work.***

We thank the reviewer for the positive assessment of our work and paper.

***The methodology was clearly presented and explained in a way that was easy to follow for a non-technical audience, and all the underlying data has been made available. It would be useful for the reader to understand in the introduction a little more about why these 5 experiments were chosen. 4 focus on sexual assault with variety over the gender of the victim and the perpetrator. The other considers interpersonal violence where there was no plausible deniability for the accused perpetrator. These are justifiable choices to test the effect of framing in different scenarios, but more clarity on the reasons behind these choices early on would be valuable.***

We appreciate this suggestion and have added a paragraph at the end of the introduction that explains the overall logic of the experimental series (lines 118-138). Specifically, we now explicitly note that the experiments were designed to systematically vary key features of the original victim framing scenario: the source of the victim label (self vs. third party; Experiment 1); the gender and sexual orientation of the protagonists (Experiments 2 and 3); the type of misconduct and social status of the alleged perpetrator (Experiments 4 and 5); and whether the facts of the case are under dispute (Experiment 5). We highlight that the first three experiments focused on sexual assault because this is a domain where victim framing is frequently deployed in public discourse (and in the original work on this topic). Varying the gender dynamics within this domain allowed us to test whether the effects depend on cultural scripts about prototypical victims of sexual assault. Experiments 4 and 5 then extended this paradigm to different scenarios (physical domestic abuse and police shooting) to assess broader generalizability.

***A footnote to explain the elements of the Dark Triad elements would be useful…***

We appreciate this suggestion. Because PLOS ONE does not allow footnotes, we have added some additional explanation at first mention in the main text of the Experiment 1 methods (lines 252-254), noting that the Dark Triad refers to three socially aversive personality traits: (1) Machiavellianism (strategic manipulation); (2) psychopathy (callousness and lack of empathy); and (3) narcissism (grandiosity and entitlement). We agree that bringing this into the text is worthwhile.

***…as would a paragraph outlining the limitations of the study. Currently the limitations are in the final section of the paper, but I would suggest bringing this into the methodology instead, rather than presenting some fantastic findings only to leave the readers’ last thoughts about the limitations of them*.**

We appreciate the spirit of this suggestion, which we understand as a concern that readers should be aware of methodological constraints before encountering the findings. However, in our experience it is standard practice in experimental psychology to present limitations in the General discussion, where they can be evaluated in light of the actual results. Placing a limitations section within the Methods, before the findings have been presented, risks priming readers to discount the results before they have had an opportunity to evaluate them. To address the concern about ending the manuscript with limitations, we have expanded the final part of the General discussion (now under the subheading “Conclusions and future directions”) to highlight the theoretical and practical implications of the findings and show how the limitations of the work motivate several exciting future directions. We believe that these additions will enable readers to better evaluate the limitations alongside the contributions of the findings.

***The results and discussion section begins with a number of technical terms which require a short, simple explanation to make them accessible to a non-technical audience (JASP, ANOVA).***

We have added brief parenthetical definitions of these terms at first use. JASP is now introduced as “an open-source statistical analysis program,” and ANOVA is now introduced as “analyses of variance (ANOVAs)—standard statistical tests for comparing group means.” We have also expanded other technical terms at first use to improve accessibility.  ***Given the huge amount of data collected and analysed, there is evidently a lot to discuss and there is a lot of evidence provided in the results section. At times this can be difficult to follow. I would encourage the authors to include a headline summary or insert box directly under the heading of each experiment in this section to make it really clear for the audience to understand what the most significant findings were in each experiment.***

We appreciate this suggestion. We took care in our revision to be clearer about the key findings of each experiment in the experiment-specific Discussion subsections of the Results and Discussion section. At the suggestion of Reviewer 2, we have also added a new figure to the General discussion (Fig 6) that depicts overall effect sizes for citers and nonciters in each experiment, which provides an accessible visual representation of our central findings. Additionally, we have added a sentence to the figure caption for each experiment (Figs 1-5) that summarizes the pattern of results for citers and nonciters.

***Expand acronyms SE when first used.***

Done. SE is now written as “standard errors (SEs)” at first use (line 402) and in the table notes.  ***When discussing experiment 4, it would be clearer to use the surname of the relevant ‘Nick’, as currently it is difficult to determine whether we are reading about the plumber or the celebrity in each mention of ‘Nick’.***

We appreciate this concern, but we believe the text already mostly addresses it: the first mention of “Nick” in the Experiment 4 results section explicitly identifies him as “Nick (Cage/Hatch), the alleged perpetrator,” establishing that “Nick” refers to the generic male character across both celebrity status conditions. All subsequent uses of “Nick” follow from this introduction, and we have reviewed the section to confirm there is no ambiguity (i.e., we never use “Nick” to refer to one individual in particular, the plumber or celebrity). That said, we have also made one additional clarification that should help readers keep track of which last names belong to which characters (lines 686-688): “Overall, participants supported Park more when she accused a stranger (Hatch) of assault than when she accused a celebrity (Cage), and supported Nick more when he was a celebrity than when he was a stranger.” (parentheses added in revised manuscript).

***Line 609 ‘Overall, participants Park more when she accused a stranger…’ – There is a word missing here.***

Good catch, thank you. The sentence has been fixed so it now reads *“Overall, participants* ***supported*** *Park more when she accused a stranger…”* ***In the discussion relating to accusing celebrities, there are some suggestions that this could indicate people may question motivations of women who accuse celebrities of crimes. Some references to other sources that discuss this here would help to support this claim.***

We appreciate this suggestion. In the updated manuscript, we have added several references to relevant literature on this point. Specifically, we now cite the following additional sources in both the Experiment 4 methods and results and discussion sections:

Knight, J. L., Giuliano, T. A., & Sanchez-Ross, M. G. (2001). Famous or infamous? The influence of celebrity status and race on perceptions of responsibility for rape. *Basic and Applied Social Psychology, 23*(3), 183–190. https://doi.org/10.1207/S15324834BASP2303_4

Nyúl, B., Kende, A., Engyel, M., & Szabó, M. (2018). Perception of a perpetrator as a successful person predicts decreased moral judgment of a rape case and labeling it as rape. *Frontiers in Psychology, 9*, 2555. https://doi.org/10.3389/fpsyg.2018.02555

Meyer, A. (2025). Blaming the victim, preserving the icon: the gendered moral work of celebrity sexual abuse scandals. *Feminist Media Studies, 25*(8), 1890–1904. https://doi.org/10.1080/14680777.2024.2392102

***There are surprisingly few references to extant literature in the results and discussion. Some references which indicate how the findings support or challenge current understandings for each experiment would strengthen this section.***

We agree that the experiment-specific Discussion subsections were relatively light on connections to the broader literature. In the revised manuscript, we have added references throughout these sections to make these connections clearer. Specifically, in Experiment 1 we now connect the finding that self- and other-framing are equally effective in this context to the literature on Truth-Default Theory (Levine, 2014). In Experiment 2, we reference work on male victimization and the resistance to recognizing men as victims/patients (Bullock & Beckson, 2011; Reynolds et al., 2020). In Experiment 3, we now cite research on attitudes towards same-sex intimate partner violence (e.g., Rolle et al., 2018). In Experiment 4, we connect the findings to the research cited above showing that fame predicts decreased responsibility attributions and moral condemnation in rape cases (Knight et al., 2001; Nyúl et al., 2018). And in Experiment 5, we highlight connections to research on how linguistic framing and ideological orientation shape evaluations of police violence (Gerber & Jackson, 2017; Park et al., 2024)

***Perhaps the ‘General Discussion’ heading would be better renamed to ‘Conclusion’ given that there is already a ‘Results and Discussion’ section.***

We appreciate this suggestion but prefer to retain “General discussion” because this section includes substantial interpretative and theoretical discussion in addition to summarizing key findings. As mentioned above, we have added a “Conclusions and future directions” subsection to the General discussion that summarizes our overall conclusions. To add further clarity, we have also added a specific “Limitations” subsection to the General discussion. The “Results and discussion” section is an umbrella heading for the experiment-specific results subsections. In our experience, this organization is common in experimental psychology.

***In the general discussion, experiment 5 is described as a racially-charged police shooting. This racially-charged element does not come across in earlier mentions of experiment 5. The authors need to add clarity as to the racial element here. Was it a mistake to include it in the general discussion, or was it racially framed? If the latter, this needs mentioning in the methods and results of experiment 5, and consideration given to whether race plays a role in the readers’ responses.***

This is an important point that we should have flagged more clearly. The names of the protagonists in Experiment 5 (Jamal Smith vs. John O’Neil) were deliberately chosen to evoke racial associations commonly present in media coverage of police violence, consistent with then-recent public discourse about White officers using force against Black civilians. However, given that we did not explicitly manipulate race or measure racial perceptions, we are careful about making strong claims. In the revised manuscript, we now preview this dimension of the experiment in the Introduction (lines 128-131) and have added several sentences to the Experiment 5 Methods section noting that the names were selected to evoke the racial dynamics commonly associated with publicized incidents of police violence, while acknowledging that we did not directly manipulate or measure participants’ racial attributions (Lines 350-357). We have also slightly revised the General discussion to refer to the “implicitly” racially-charged nature of the scenario rather than presenting it as a definitive racial manipulation (Lines 827-829). This is consistent with the more detailed treatment of this point in the Supplemental Material (S1 Text; Experiment 5 pilot study section).

***There is a really interesting discussion of the age and political persuasion of the participants and how these might influence their responses. Adding references to wider literature that discusses how these elements may influence individuals’ approaches to crimes and the parties involve would be valuable and relevant.***

Thank you for the suggestion. We have now added the following references to this part of the General Discussion, citing (a) research on the relationship between political ideology and attitudes towards crime and punishment, and (b) generational differences in attitudes towards sexual assault and consent (line 857).

Nagel, B., Matsuo, H., McIntyre, K. P., & Morrison, N. (2005). Attitudes toward victims of rape: Effects of gender, race, religion, and social class. *Journal of Interpersonal Violence, 20*(6), 725–737. https://doi.org/10.1177/08862605052760

Silver, J. R., & Silver, E. (2017). Why are conservatives more punitive than liberals? A moral foundations approach. *Law and Human Behavior, 41*(3), 258–272. https://doi.org/10.1037/lhb0000232

***Indications of future research plans that will build on or expand these experiments would be very valuable as a final round off of the piece.***

We appreciate this suggestion. In the revised manuscript, we have expanded the final paragraph of the General Discussion (under new subheading “Conclusions & Future Directions”) to include more concrete suggestions for future research (lines 958-972):

*“Looking ahead, several lines of future research would strengthen and extend the present findings. First, directly manipulating pragmatic cues such as source credibility would help test whether the effects of victim framing track predictions of the social-pragmatic account specifically. Relatedly, future work could assess individual differences in pragmatic sensitivity to victim labeling and examine whether such sensitivity predicts the magnitude of victim framing effects, as in recent work on grammatical framing [44]. This would provide a more direct test of the role of pragmatic inference in victim framing than the post-hoc citer classification used here. Additionally, a longitudinal study design with multiple sessions could test whether the modest victim framing effects we observed accumulate or attenuate with repeated exposure to the framing narrative. Finally, extending the paradigm to other languages and non-U.S. samples would help determine whether victim framing operates similarly in cultural contexts where norms about victimhood, authority, and gender differ. Ongoing work in our labs is beginning to address several of these questions. In the meantime, the present findings serve as a reminder to all who communicate about morally charged issues: language choices matter, even seemingly small ones.”*

***Reviewer #2: Strengths: This manuscript presents a rigorous, large-scale experimental investigation of victim framing across five diverse social scenarios, using well-controlled vignette manipulations, substantial sample sizes, preregistration, and open data practices. The multi-study design provides strong internal validity and valuable evidence that explicit victim labeling can systematically influence moral and social judgments beyond prototypical sexual assault cases. The inclusion of baseline conditions, multiple participant pools, and theoretically relevant individual difference measures further strengthens the empirical foundation and enhances the credibility and scope of the findings.***

Thank you for the kind words about the manuscript.

***The manuscript would benefit from moderating its claims about the underlying psychological mechanism, particularly regarding social-pragmatic inference, since key moderator variables are measured post hoc rather than experimentally manipulated. Clarifying conceptual definitions of victim framing, acknowledging modest effect sizes and ecological limits of vignette methods, and streamlining repetitive methodological sections would also improve clarity and theoretical precision. Adding a concise limitations section and slightly tempering generalizability claims would make the argument more balanced and persuasive to a broad interdisciplinary readership.***

We appreciate these comments and have updated the manuscript to address them all. We provide additional details in response to the specific comments below.  ***1. Clearly distinguish between explicit victim labeling (the focus of the current studies) and broader narrative or structural victim positioning. This will prevent overgeneralization and situate the work as a precise test of lexical framing rather than all forms of moral positioning.***

This is a valuable distinction; thank you for highlighting it. We have added the term “explicit” to the phrase “victim framing” in the Abstract and Introduction to mark this distinction, as well as throughout the paper, and return to this issue in more detail in our General Discussion, where it now features prominently in the limitations section (lines 909-917):

*“Relatedly, we only tested the effects of explicit victim framing, where the word “victim” was directly applied to a specific individual. This is distinct from general narrative strategies that position someone as a victim through selective emphasis, tone, or other subtle elements of a story without ever using the label itself. We focused on the explicit case because it is a common and recognizable form of the victim framing strategy [2] and is more easily investigated using established methods in the linguistic framing literature [11]. Although subtler forms of victim framing may also shape moral evaluations, this remains to be tested empirically. It is possible that using the victim label explicitly led our participants to become more attuned to victim framing than they would be in real-world media environments.”*

***2. The interpretation that effects arise from social-pragmatic inference is plausible but not directly tested. Reframe this as a theoretically consistent explanation rather than a demonstrated mechanism, and acknowledge alternatives such as motivated reasoning or heuristic processing.***

We agree that this is an important distinction and have revised the manuscript accordingly throughout. The Abstract now describes the findings as “consistent with” a social-pragmatic account rather than directly demonstrating it. This language is carried over throughout the Introduction, Results and Discussion, and General Discussion sections. For example, in the Introduction we now acknowledge that other mechanisms (e.g., dyadic moral reasoning) may also play a role (lines 107-108). In the General Discussion, we have added several sentences highlighting these alternative mechanisms (lines 835-846):

*“That being said, the measure of “citing” victim-related language was a post-hoc, self-report indicator of attention to the framing language rather than a direct measure of pragmatic inference, and several additional mechanisms may also help explain these findings. For example, the victim label may have led to “moral typecasting” [34], activating a moral agent/patient schema that constrained subsequent evaluations. As moral patients, victims are generally viewed as less capable of inflicting moral harm. The victim label could also function as a heuristic cue that shortcuts more effortful evaluations of the protagonists given its general cultural connotations. And confirmation bias could selectively direct attention to evidence in the narrative consistent with the labeled individual’s victimhood. These explanations are not mutually exclusive with the social-pragmatic account. In fact, pragmatic inference might serve as the initial mechanism that activates one or more downstream processes, though future research is needed to distinguish among possible mechanistic pathways.”*

***3. While framing effects appear across multiple contexts, their magnitude and consistency vary. Distinguish between demonstrating presence of effects and demonstrating equivalent psychological operation across scenarios.***

We appreciate this point and have highlighted this distinction in the General Discussion when we summarize the findings (lines 820-824):

*“There was some variability in the magnitude of these effects and in baseline support for the protagonists across contexts, suggesting that victim framing does not operate identically in all scenarios. Notably, however, participants who explicitly cited the victim-related language as influencing their evaluations showed robust framing effects in all contexts, expressing elevated support for the victim-framed individual.”*

***4. Because the manipulation uses explicit victim terminology, participants may become aware of the linguistic emphasis. Acknowledge that this may increase salience relative to real-world, subtler framing.***

We have added this acknowledgment to the limitations section of the General Discussion (see our response to comment #1 above for updated text).

***5. The baseline condition lacks explicit victim language but is not psychologically neutral. Readers may still rely on cultural scripts. Clarify that this is a no-label control, not a value-free reference point.***

This is a great point. In the Methods section, we now flag this issue directly (lines 183-188):

*“The baseline condition served as a no-label control since neither individual was described using victim-related language. Importantly, however, participants in this condition were not in a psychologically neutral state: they brought their own cultural scripts, prior beliefs, and moral convictions to bear on the same scenario as those in the victim framing conditions. The baseline condition therefore provides a reference point for evaluating the incremental impact of explicit victim framing in shaping judgments in these cases.”*

***6. The vignettes are necessarily simplified. Real-world judgments involve legal norms, historical context, media repetition, and social identity cues. A short paragraph acknowledging these limits would strengthen interpretive balance.***

We appreciate this point and have now expanded the Limitations section to better acknowledge these elements of the framing landscape (lines 899-938).

***7. The composite outcome combines empathy, credibility, harm, and responsibility. If possible, report whether framing affects these components differently, as they map onto distinct psychological processes.***

This is an interesting suggestion. We used the composite scores to remain consistent with prior work on victim framing (Flusberg et al., 2022) and because the components showed good internal consistency (Cronbach’s ɑ = 0.70–0.85). Analyzing a single composite score as opposed to four separate measures also reduces the chances of committing a Type 1 error. However, we appreciate the theoretical interest in disaggregating the components. We have added these analyses to the Supplemental Material (S1 text) and have included a new supplemental table to display our findings. Here is the updated supplemental text:

*“Overall, the frame × citer interaction was significant or trending for the majority of component measures, and the effects were consistently in the same direction as the composite measure. In Experiment 2, the interaction was significant for every component for both the accuser and alleged perpetrator, closely mirroring the composite results. In Experiment 4, the interaction was significant for all components except believability of the alleged perpetrator. However, we did observe some variation across components. For example, in Experiment 1, the interaction was significant for empathy, believability, and harm for the accuser, but not responsibility, and was nonsignificant for all components except believability for the alleged perpetrator. In Experiment 3, the interaction was significant only for believability (accuser) and empathy and believability (alleged perpetrator). In Experiment 5, the interaction was significant for empathy and responsibility but not suffering for both protagonists. Taken together, these patterns suggest that the composite score provides a reasonable summary of individual component framing effects, but that explicit victim framing may most reliably affect perceptions of empathy and believability, with more variable impacts on perceived harm/emotional suffering and responsibility.”*

***8. Clarify that citer status is a post-exposure self-report indicator of attention to framing language, not a clean measure of pragmatic inference. This avoids overinterpreting it as a causal moderator.***

We have now done this in the General Discussion section (see response to Comment 2 above). We have also added the following line to the Methods section where we introduce the citing measure (lines 218-221):

*“As in prior work on victim framing [2], we used this citer/nonciter distinction as an indirect marker of pragmatic sensitivity to the victim framing language, though it does not directly assess the underlying inferential process.”*

***9. Explain whether measures like rape myth acceptance, political ideology, or police legitimacy are intended as moderators of framing susceptibility or simply covariates. This will improve conceptual coherence.***

We have clarified this in the revised Methods and Results and Discussion sections. The individual difference measures were included primarily as covariates to assess whether victim framing effects persist above and beyond individual differences in preexisting attitudes and demographics. These analyses were largely exploratory (and indeed were preregistered as such), which is why they are included in the Supplemental Material (S1 Text) rather than the main manuscript. We now state the purpose of including these measures explicitly in the Overall Approach subsection (lines 225-228):

*“These measures enabled us to assess whether preexisting attitudes predict support for the protagonists in expected ways, and whether victim framing effects persist above and beyond such attitudes, though these analyses were preregistered as exploratory (see S1 Text).”*

***10. Many effects are statistically significant but modest. Briefly note that small-to-moderate effects are typical in framing research, which contextualizes the findings appropriately.***

We have added a couple of sentences to the General Discussion (lines 879-884) contextualizing the effect sizes from our studies, which are also depicted in the new Fig 6 (see our response to comment #16 below):

*“And among citers, effect sizes for victim framing were mostly in the moderate range (Cohen’s ds between 0.50 and 0.88), consistent with the broader linguistic framing literature [11]. See Fig 6. The exception was Experiment 4, the celebrity domestic violence case, where effect sizes were notably larger (Cohen’s d = 1.24 and 1.28). This may reflect the relative sparseness of factual details in the Experiment 4 vignette, which gave the victim label more room to influence evaluations in the absence of competing information.”*

***11. Make clearer which analyses were preregistered versus exploratory, especially in sections involving covariates and individual differences.***

We have revised the Overall Approach subsection at the beginning of the Results and Discussion section to more clearly delineate which analyses were preregistered and which were exploratory. Specifically, the central preregistered analyses were the ANOVAs assessing overall framing effects and the frame × citer status interactions. All analyses including individual difference measures and demographics as covariates were exploratory (though we did note we might conduct these exploratory analyses *as* exploratory analyses in our preregistrations).

***12. Replace strong causal wording about pragmatic inference with more cautious phrasing.***

We have done this throughout the manuscript (see our response to comment #2 for additional details). We have replaced causal language (e.g., “demonstrates,” “confirms”) with appropriately hedged/cautious alternatives (e.g., “is consistent with,” “aligns with,” “suggests”).

***13. Include explicit discussion of fictional scenarios, Explicit (not subtle) framing, Post hoc citer classification, U.S.-only samples, and modest effect sizes.***

Each of these points is now explicitly addressed in the revised General Discussion, as detailed in our responses to the other comments on these topics.

***14. When discussing media, legal, or political implications, emphasize that real-world communication involves additional factors (source credibility, repetition, visual media) not captured here.***

We have added qualifying language to the final section of the General Discussion, where the implications of this work are discussed (lines 953-957):

*“That said, real-world communication about allegations of wrongdoing typically includes narrative repetition, visual and social media, source credibility information, and contextual factors that our studies could not capture. Therefore, our findings should be understood as demonstrating the potential influence of one specific linguistic strategy within a broader communicative ecosystem.”*

***15. The author should condense repeated procedural details across experiments into a shared section to improve readability and flow.***

We appreciate the suggestion, which we did our best to implement in the original manuscript. After careful review, we do not believe there are any additional shared or repeated procedural details that can be moved to the General Design and Procedure subsection.

***16. A visual overview of effect directions and strengths across all five experiments would help readers quickly grasp the overall pattern.***

This is a great suggestion. We have added a new figure (Fig 6) to the General discussion that provides a summary of victim framing effects across all five experiments, as indicated by Cohen’s *d* values reflecting the contrast between the two active framing conditions (assault victim vs. allegation victim in Experiments 1-4; civilian victim vs. police victim in Experiment 5), computed separately for citers and nonciters. The figure depicts these effect sizes on support scores for the accuser/civilian (left panel) and the alleged perpetrator/police officer (right panel). Across all five experiments, citers showed robust and consistent framing effects—though effect sizes were variable—while nonciters showed minimal differentiation between conditions.

***17. The authors briefly acknowledge that effects might also reflect moral typecasting, schema activation, or confirmation bias, not only pragmatic inference.***

We have added this acknowledgment to the General Discussion, as detailed in our response to comment #2 above.

***18. In some cases framing differences are small or non-significant. Note whether this reflects contextual resistance to framing or simply reduced statistical power.***

This is a good point, thank you. It is not always clear how to interpret small or nonsignificant results in studies like these, but we have added several notes in the relevant experiment-specific results sections to address this issue.

For example, for Experiment 1, we have now added the following after reporting no significant frame × citer status interaction (lines 441-443): “*The absence of a significant interaction likely reflects the fact that both citers and nonciters showed some sensitivity to the framing of the alleged perpetrator, leaving less room for a divergence to emerge.”*

In Experiment 2, after reporting no main effect on support for the male accuser, we now include the following explanation (lines 528-531): *“This nonsignificant effect on support for David, the male accuser, could reflect cultural resistance to recognizing men as victims of sexual assault [25], reduced statistical power for detecting accuser effects in this counter-stereotypical scenario, or both.”*

In Experiment 3 (same-sex assault), we now note (lines 656-659): “*In light of these factors, the attenuation of victim framing effects in cases involving two women may be due to contextual resistance to the framing manipulation. However, it could also be an artifact of reduced statistical power within the gender subconditions or simply a statistical anomaly.”*

***19. Please, propose designs if possible that manipulate pragmatic cues directly, use process-tracing methods, or measure real-time interpretation to better isolate cognitive mechanisms.***

We appreciate this suggestion, though we note that it is difficult in practice to conduct framing studies using intensive lab procedures or qualitative methods since the sample sizes required are so large. However, we now highlight the need for more direct manipulations of pragmatic cues and direct measures of pragmatic inference in the final paragraph of our revised General Discussion, in the new Conclusions and Future Directions subsection (lines 958-964):

*“Looking ahead, several lines of future research would strengthen and extend the present findings. First, directly manipulating pragmatic cues such as source credibility would help test whether the effects of victim framing track predictions of the social-pragmatic account specifically. Relatedly, future work could assess individual differences in pragmatic sensitivity to victim labeling and examine whether such sensitivity predicts the magnitude of victim framing effects, as in recent work on grammatical framing [44]. This would provide a more direct test of the role of pragmatic inference in victim framing than the post-hoc citer classification used here.”*

***20. In the discussion, succinctly restate that the main empirical contribution is demonstrating cross-context robustness of victim labeling effects, independent of specific content domains.***

We appreciate this suggestion. The following now appears at the beginning of the General Discussion (lines 811-814):

*“The central empirical contribution of the present set of studies is to demonstrate that the effects of victim framing generalize across a diverse range of scenarios, providing evidence that this linguistic strategy operates in many contexts, not just in prototypical sexual assault cases.”*

***21. Future work could test whether victim framing operates similarly in non-U.S. contexts where norms about victimhood, authority, and gender differ.***

This point was briefly noted in the original limitations section, but we have expanded it into a more substantive call for cross-cultural research in our revised Conclusions and Future Directions subsection (lines 967-970):

*“Finally, extending the paradigm to other languages and non-U.S. samples would help determine whether victim framing operates similarly in cultural contexts where norms about victimhood, authority, and gender differ. Ongoing work in our labs is beginning to address several of these questions.”*

***22. Even small effects in framing can have large societal consequences when repeated in media environments. A brief note on cumulative impact would help contextualize importance.***

This is a good point, and one we have made in previous work on linguistic framing effects. We have added a sentence in the revised General Discussion to this effect, in the section where we discuss effect sizes (lines 884-886):

*“That said, even modest effects can compound when framing language is repeated across millions of media exposures, potentially shaping public opinion about even hotly debated topics.”*

***23. The author must ensure consistent use of terms such as “victim framing,” “victim labeling,” and “victim language” to avoid conceptual drift.***

Thank you for the reminder. We have conducted a careful audit of the manuscript to ensure that we consistently use “victim framing” as the overarching term for the rhetorical strategy, “victim label” or “victim labeling” when referring specifically to the explicit use of the word “victim,” and “victim-related language” when referring to the broader set of terms used in the manipulation (victim, traumatized, survive, etc.).

***24. Where boxplots are used, consider adding brief interpretive captions summarizing the key takeaway from each figure.***

This is a nice suggestion, thank you. We have revised all figure captions to include a brief interpretative statement following the overview. For example, the caption for Fig 1 now concludes with: *“Citers showed the expected framing effect on support and Dark Triad judgments of the accuser and alleged perpetrator alike, while nonciters showed only a small framing effect on support for the alleged perpetrator.”*

***25. Please, end with a concise synthesis connecting linguistic framing, moral cognition, and social judgment, reinforcing how this work bridges these domains.***

We have revised the final part of the General Discussion (now titled “Conclusions and future directions”) to provide this synthesis, though we positioned it before our call for future research and our final conclusion statement (lines 939-957):

*“Despite its limitations, the present research has important implications for understanding the relationships among linguistic framing, social cognition, and moral reasoning. Victimhood has become central to political and moral debate, with competing groups claiming victim status to elevate their grievances and delegitimize their opponents [41]. Our results show that explicit victim framing is more than a rhetorical flourish: across five diverse scenarios, the victim label genuinely shaped how a substantial proportion of people evaluated moral disputes, even when controlling for preexisting attitudes that strongly predicted evaluations on their own. These findings sit at the intersection of three broader themes. First, they add to a growing literature on the power of basic lexical choices to influence attitudes and decision-making [11]. Second, they align with the view that pragmatic inference plays a key role in how people interpret language and, consequently, how subtle linguistic choices shape social judgments [2, 9-11, 44]. And third, they carry practical stakes: understanding when, how, and why labeling someone a victim shapes observer reactions is directly relevant to journalists, advocates, and policymakers who discuss morally charged issues. That said, real-world communication about allegations of wrongdoing typically includes narrative repetition, visual and social media, source credibility information, and contextual factors that our studies could not capture. Therefore, our findings should be understood as demonstrating the potential influence of one specific linguistic strategy within a broader communicative ecosystem.”*
