# Supplementary material for: Victim framing shapes attitudes across diverse contexts
Source: PLoS One. 2026 Jun 12;21(6):e0351416. doi: 10.1371/journal.pone.0351416 (PMC13262844; doi:10.1371/journal.pone.0351416)
Supplement: Peer review history

# Peer review: Decision Letter 0

**DOI:** 10.1371/journal.pone.0351416.r001

**Authors:** Jenna Scaramanga

26 Feb 2026

PONE-D-25-65711

Victim framing shapes attitudes across diverse contexts

PLOS One

Dear Dr. Flusberg,

Thank you for submitting your manuscript to PLOS ONE. After careful consideration, we feel that it has merit but does not fully meet PLOS ONE’s publication criteria as it currently stands. Therefore, we invite you to submit a revised version of the manuscript that addresses the points raised during the review process.

Your submission has been evaluated by two reviewers, and their comments are appended below. Please carefully revise your manuscript to address the points raised.

Please submit your revised manuscript by Mar 30 2026 11:59PM. If you will need more time than this to complete your revisions, please reply to this message or contact the journal office at plosone@plos.org. When you're ready to submit your revision, log on to https://www.editorialmanager.com/pone/ and select the 'Submissions Needing Revision' folder to locate your manuscript file.

Please include the following items when submitting your revised manuscript:

- A letter that responds to each point raised by the academic editor and reviewer(s). You should upload this letter as a separate file labeled 'Response to Reviewers'.
- A marked-up copy of your manuscript that highlights changes made to the original version. You should upload this as a separate file labeled 'Revised Manuscript with Track Changes'.
- An unmarked version of your revised paper without tracked changes. You should upload this as a separate file labeled 'Manuscript'.

If you would like to make changes to your financial disclosure, please include your updated statement in your cover letter. Guidelines for resubmitting your figure files are available below the reviewer comments at the end of this letter.

If applicable, we recommend that you deposit your laboratory protocols in protocols.io to enhance the reproducibility of your results. Protocols.io assigns your protocol its own identifier (DOI) so that it can be cited independently in the future. For instructions see: https://journals.plos.org/plosone/s/submission-guidelines#loc-laboratory-protocols. Additionally, PLOS ONE offers an option for publishing peer-reviewed Lab Protocol articles, which describe protocols hosted on protocols.io. Read more information on sharing protocols at https://plos.org/protocols?utm_medium=editorial-email&utm_source=authorletters&utm_campaign=protocols.

We look forward to receiving your revised manuscript.

Kind regards,

Jenna Scaramanga

Staff Editor

PLOS One

Journal Requirements:

When submitting your revision, we need you to address these additional requirements.

1. Please ensure that your manuscript meets PLOS ONE's style requirements, including those for file naming. The PLOS ONE style templates can be found at

https://journals.plos.org/plosone/s/file?id=wjVg/PLOSOne_formatting_sample_main_body.pdf and

https://journals.plos.org/plosone/s/file?id=ba62/PLOSOne_formatting_sample_title_authors_affiliations.pdf.

2. Thank you for stating the following in the Acknowledgments Section of your manuscript:

“Experiments 1 and 5 were funded by the Galakatos Science Research Fund at Reed College. Experiments 2 and 4 were funded by the senior thesis support funding at SUNY Purchase College. Experiment 3 was funded by Vassar startup funds awarded to SJF.”

We note that you have provided additional information within the Acknowledgements Section that is not currently declared in your Funding Statement. Please note that funding information should not appear in the Acknowledgments section or other areas of your manuscript. We will only publish funding information present in the Funding Statement section of the online submission form.

Please remove any funding-related text from the manuscript and let us know how you would like to update your Funding Statement. Currently, your Funding Statement reads as follows:

“The author(s) received no specific funding for this work.”

Please include your amended statements within your cover letter; we will change the online submission form on your behalf.

3. Please include your full ethics statement in the ‘Methods’ section of your manuscript file. In your statement, please include the full name of the IRB or ethics committee who approved or waived your study, as well as whether or not you obtained informed written or verbal consent. If consent was waived for your study, please include this information in your statement as well.

4. We note that there is identifying data in the Supporting Information file < Supplemental Material.docx>. Due to the inclusion of these potentially identifying data, we have removed this file from your file inventory. Prior to sharing human research participant data, authors should consult with an ethics committee to ensure data are shared in accordance with participant consent and all applicable local laws.

Data sharing should never compromise participant privacy. It is therefore not appropriate to publicly share personally identifiable data on human research participants. The following are examples of data that should not be shared:

-Name, initials, physical address

-Ages more specific than whole numbers

-Internet protocol (IP) address

-Specific dates (birth dates, death dates, examination dates, etc.)

-Contact information such as phone number or email address

-Location data

-ID numbers that seem specific (long numbers, include initials, titled “Hospital ID”) rather than random (small numbers in numerical order)

Data that are not directly identifying may also be inappropriate to share, as in combination they can become identifying. For example, data collected from a small group of participants, vulnerable populations, or private groups should not be shared if they involve indirect identifiers (such as sex, ethnicity, location, etc.) that may risk the identification of study participants.

Additional guidance on preparing raw data for publication can be found in our Data Policy (https://journals.plos.org/plosone/s/data-availability#loc-human-research-participant-data-and-other-sensitive-data) and in the following article: http://www.bmj.com/content/340/bmj.c181.long.

Please remove or anonymize all personal information (<specific identifying information in file to be removed>), ensure that the data shared are in accordance with participant consent, and re-upload a fully anonymized data set. Please note that spreadsheet columns with personal information must be removed and not hidden as all hidden columns will appear in the published file.

5. If the reviewer comments include a recommendation to cite specific previously published works, please review and evaluate these publications to determine whether they are relevant and should be cited. There is no requirement to cite these works unless the editor has indicated otherwise.

[Note: HTML markup is below. Please do not edit.]

Reviewers' comments:

Reviewer's Responses to Questions

**Comments to the Author**

1. Is the manuscript technically sound, and do the data support the conclusions?

The manuscript must describe a technically sound piece of scientific research with data that supports the conclusions. Experiments must have been conducted rigorously, with appropriate controls, replication, and sample sizes. The conclusions must be drawn appropriately based on the data presented.

Reviewer #1: Yes

Reviewer #2: Partly

**********

2. Has the statistical analysis been performed appropriately and rigorously?

Reviewer #1: I Don't Know

Reviewer #2: Yes

**********

3. Have the authors made all data underlying the findings in their manuscript fully available?

The PLOS Data policy requires authors to make all data underlying the findings described in their manuscript fully available without restriction, with rare exception (please refer to the Data Availability Statement in the manuscript PDF file). The data should be provided as part of the manuscript or its supporting information, or deposited to a public repository. For example, in addition to summary statistics, the data points behind means, medians and variance measures should be available. If there are restrictions on publicly sharing data—e.g. participant privacy or use of data from a third party—those must be specified.

Reviewer #1: Yes

Reviewer #2: Yes

**********

4. Is the manuscript presented in an intelligible fashion and written in standard English?

PLOS ONE does not copyedit accepted manuscripts, so the language in submitted articles must be clear, correct, and unambiguous. Any typographical or grammatical errors should be corrected at revision, so please note any specific errors here.

Reviewer #1: Yes

Reviewer #2: Yes

**********

5. Review Comments to the Author

Please use the space provided to explain your answers to the questions above. You may also include additional comments for the author, including concerns about dual publication, research ethics, or publication ethics. (Please upload your review as an attachment if it exceeds 20,000 characters)

Reviewer #1: This is an interesting piece on the weight of victim framing in how people understand perpetrators of offences.

It follows up on a recent set of studies on victim framing which looked at men accused of sexual assault where respondents who cited victim-related language as influential believed that the victim language was included because that person deserved their support. This study expands this research to look at contexts beyond accusations of men sexually assaulting women to determine whether the linguistic victim framing applies in different contexts.

5 experiments were conducted, each considering a different type of case. A total of 3,057 participants were sampled, with 2,941 analyzed (with reasons for the decrease explained).

Each experiment has so much data that there could very well be enough content for a paper on each one, but this article does a good job of highlighting the key features and key findings of the experiment as a whole.

The introduction is clearly presented and offers useful context for the work.

The methodology was clearly presented and explained in a way that was easy to follow for a non-technical audience, and all the underlying data has been made available. It would be useful for the reader to understand in the introduction a little more about why these 5 experiments were chosen. 4 focus on sexual assault with variety over the gender of the victim and the perpetrator. The other considers interpersonal violence where there was no plausible deniability for the accused perpetrator. These are justifiable choices to test the effect of framing in different scenarios, but more clarity on the reasons behind these choices early on would be valuable. A footnote to explain the elements of the Dark Triad elements would be useful, as would a paragraph outlining the limitations of the study. Currently the limitations are in the final section of the paper, but I would suggest bringing this into the methodology instead, rather than presenting some fantastic findings only to leave the readers’ last thoughts about the limitations of them.

The results and discussion section begins with a number of technical terms which require a short, simple explanation to make them accessible to a non-technical audience (JASP, ANOVA).

Given the huge amount of data collected and analysed, there is evidently a lot to discuss and there is a lot of evidence provided in the results section. At times this can be difficult to follow. I would encourage the authors to include a headline summary or insert box directly under the heading of each experiment in this section to make it really clear for the audience to understand what the most significant findings were in each experiment.

Expand acronyms SE when first used.

When discussing experiment 4, it would be clearer to use the surname of the relevant ‘Nick’, as currently it is difficult to determine whether we are reading about the plumber or the celebrity in each mention of ‘Nick’.

Line 609 ‘Overall, participants Park more when she accused a stranger…’ – There is a word missing here.

In the discussion relating to accusing celebrities, there are some suggestions that this could indicate people may question motivations of women who accuse celebrities of crimes. Some references to other sources that discuss this here would help to support this claim.

There are surprisingly few references to extant literature in the results and discussion. Some references which indicate how the findings support or challenge current understandings for each experiment would strengthen this section.

Perhaps the ‘General Discussion’ heading would be better renamed to ‘Conclusion’ given that there is already a ‘Results and Discussion’ section.

In the general discussion, experiment 5 is described as a racially-charged police shooting. This racially-charged element does not come across in earlier mentions of experiment 5. The authors need to add clarity as to the racial element here. Was it a mistake to include it in the general discussion, or was it racially framed? If the latter, this needs mentioning in the methods and results of experiment 5, and consideration given to whether race plays a role in the readers’ responses.

There is a really interesting discussion of the age and political persuasion of the participants and how these might influence their responses. Adding references to wider literature that discusses how these elements may influence individuals’ approaches to crimes and the parties involve would be valuable and relevant.

Indications of future research plans that will build on or expand these experiments would be very valuable as a final round off of the piece.

Reviewer #2: Strengths: This manuscript presents a rigorous, large-scale experimental investigation of victim framing across five diverse social scenarios, using well-controlled vignette manipulations, substantial sample sizes, preregistration, and open data practices. The multi-study design provides strong internal validity and valuable evidence that explicit victim labeling can systematically influence moral and social judgments beyond prototypical sexual assault cases. The inclusion of baseline conditions, multiple participant pools, and theoretically relevant individual difference measures further strengthens the empirical foundation and enhances the credibility and scope of the findings.

The manuscript would benefit from moderating its claims about the underlying psychological mechanism, particularly regarding social-pragmatic inference, since key moderator variables are measured post hoc rather than experimentally manipulated. Clarifying conceptual definitions of victim framing, acknowledging modest effect sizes and ecological limits of vignette methods, and streamlining repetitive methodological sections would also improve clarity and theoretical precision. Adding a concise limitations section and slightly tempering generalizability claims would make the argument more balanced and persuasive to a broad interdisciplinary readership.

1. Clearly distinguish between explicit victim labeling (the focus of the current studies) and broader narrative or structural victim positioning. This will prevent overgeneralization and situate the work as a precise test of lexical framing rather than all forms of moral positioning.

2. The interpretation that effects arise from social-pragmatic inference is plausible but not directly tested. Reframe this as a theoretically consistent explanation rather than a demonstrated mechanism, and acknowledge alternatives such as motivated reasoning or heuristic processing.

3. While framing effects appear across multiple contexts, their magnitude and consistency vary. Distinguish between demonstrating presence of effects and demonstrating equivalent psychological operation across scenarios.

4. Because the manipulation uses explicit victim terminology, participants may become aware of the linguistic emphasis. Acknowledge that this may increase salience relative to real-world, subtler framing.

5. The baseline condition lacks explicit victim language but is not psychologically neutral. Readers may still rely on cultural scripts. Clarify that this is a no-label control, not a value-free reference point.

6. The vignettes are necessarily simplified. Real-world judgments involve legal norms, historical context, media repetition, and social identity cues. A short paragraph acknowledging these limits would strengthen interpretive balance.

7. The composite outcome combines empathy, credibility, harm, and responsibility. If possible, report whether framing affects these components differently, as they map onto distinct psychological processes.

8. Clarify that citer status is a post-exposure self-report indicator of attention to framing language, not a clean measure of pragmatic inference. This avoids overinterpreting it as a causal moderator.

9. Explain whether measures like rape myth acceptance, political ideology, or police legitimacy are intended as moderators of framing susceptibility or simply covariates. This will improve conceptual coherence.

10. Many effects are statistically significant but modest. Briefly note that small-to-moderate effects are typical in framing research, which contextualizes the findings appropriately.

11. Make clearer which analyses were preregistered versus exploratory, especially in sections involving covariates and individual differences.

12. Replace strong causal wording about pragmatic inference with more cautious phrasing.

13. Include explicit discussion of fictional scenarios, Explicit (not subtle) framing, Post hoc citer classification, U.S.-only samples, and modest effect sizes.

14. When discussing media, legal, or political implications, emphasize that real-world communication involves additional factors (source credibility, repetition, visual media) not captured here.

15. The author should condense repeated procedural details across experiments into a shared section to improve readability and flow.

16. A visual overview of effect directions and strengths across all five experiments would help readers quickly grasp the overall pattern.

17. The author sriefly acknowledge that effects might also reflect moral typecasting, schema activation, or confirmation bias, not only pragmatic inference.

18. In some cases framing differences are small or non-significant. Note whether this reflects contextual resistance to framing or simply reduced statistical power.

19. Please, propose designs if possible that manipulate pragmatic cues directly, use process-tracing methods, or measure real-time interpretation to better isolate cognitive mechanisms.

20. In the discussion, succinctly restate that the main empirical contribution is demonstrating cross-context robustness of victim labeling effects, independent of specific content domains.

21. Future work could test whether victim framing operates similarly in non-U.S. contexts where norms about victimhood, authority, and gender differ.

22. Even small effects in framing can have large societal consequences when repeated in media environments. A brief note on cumulative impact would help contextualize importance.

23. The author must ensure consistent use of terms such as “victim framing,” “victim labeling,” and “victim language” to avoid conceptual drift.

24. Where boxplots are used, consider adding brief interpretive captions summarizing the key takeaway from each figure.

25. Please, end with a concise synthesis connecting linguistic framing, moral cognition, and social judgment, reinforcing how this work bridges these domains.

**********

6. PLOS authors have the option to publish the peer review history of their article (what does this mean?). If published, this will include your full peer review and any attached files.

If you choose “no”, your identity will remain anonymous but your review may still be made public.

**Do you want your identity to be public for this peer review?** For information about this choice, including consent withdrawal, please see our Privacy Policy.

Reviewer #1: No

Reviewer #2: No

**********

[NOTE: If reviewer comments were submitted as an attachment file, they will be attached to this email and accessible via the submission site. Please log into your account, locate the manuscript record, and check for the action link "View Attachments". If this link does not appear, there are no attachment files.]

To ensure your figures meet our technical requirements, please review our figure guidelines: https://journals.plos.org/plosone/s/figures

You may also use PLOS’s free figure tool, NAAS, to help you prepare publication quality figures: https://journals.plos.org/plosone/s/figures#loc-tools-for-figure-preparation.

NAAS will assess whether your figures meet our technical requirements by comparing each figure against our figure specifications.

# Author comment: Author response to Decision Letter 1

**DOI:** 10.1371/journal.pone.0351416.r002

31 Mar 2026

We have carefully addressed all reviewer comments and journal requirements. Amongst other changes, we have tempered our claims about the social-pragmatic account of victim framing, clarified our rationale for the experiments and the implicit racial dimension of Experiment 5, included a new figure that provides a summary of effect sizes across all studies, added additional references and connections to existing research throughout, clarified key terms and the nature of our findings, and provided an expanded General discussion that enhances our treatment of study limitations and discusses future research directions. We have also taken this opportunity to correct minor issues throughout the manuscript for improved readability.

Regarding the journal requirements specified by the editor:

● We have formatted the revised manuscript to meet PLOS ONE style requirements, including file naming conventions, headings and subheadings, figure labeling, references style, and more.

● We have removed funding information from the Acknowledgments section. Please update the Funding Statement to read: "Experiments 1 and 5 were funded by the Galakatos Science Research Fund at Reed College. Experiments 2 and 4 were funded by the senior thesis support funding at SUNY Purchase College. Experiment 3 was funded by Vassar startup funds awarded to SJF."

● We have added a full ethics statement to the Methods section, including the names of the IRBs that approved each experiment and confirmation that informed written consent was obtained from all participants.

● We have removed all potentially identifying information (e.g., specific dates, timestamps) from the data files on OSF and the supporting information file.

A detailed, point-by-point response to all reviewer comments is provided in the accompanying "response to reviewers" document and pasted below:

Response to Reviewers

5. Review Comments to the Author

Please use the space provided to explain your answers to the questions above. You may also include additional comments for the author, including concerns about dual publication, research ethics, or publication ethics. (Please upload your review as an attachment if it exceeds 20,000 characters)

Reviewer #1: This is an interesting piece on the weight of victim framing in how people understand perpetrators of offences. It follows up on a recent set of studies on victim framing which looked at men accused of sexual assault where respondents who cited victim-related language as influential believed that the victim language was included because that person deserved their support. This study expands this research to look at contexts beyond accusations of men sexually assaulting women to determine whether the linguistic victim framing applies in different contexts. 5 experiments were conducted, each considering a different type of case. A total of 3,057 participants were sampled, with 2,941 analyzed (with reasons for the decrease explained). Each experiment has so much data that there could very well be enough content for a paper on each one, but this article does a good job of highlighting the key features and key findings of the experiment as a whole. The introduction is clearly presented and offers useful context for the work.

We thank the reviewer for the positive assessment of our work and paper.

The methodology was clearly presented and explained in a way that was easy to follow for a non-technical audience, and all the underlying data has been made available. It would be useful for the reader to understand in the introduction a little more about why these 5 experiments were chosen. 4 focus on sexual assault with variety over the gender of the victim and the perpetrator. The other considers interpersonal violence where there was no plausible deniability for the accused perpetrator. These are justifiable choices to test the effect of framing in different scenarios, but more clarity on the reasons behind these choices early on would be valuable.

We appreciate this suggestion and have added a paragraph at the end of the introduction that explains the overall logic of the experimental series (lines 118-138). Specifically, we now explicitly note that the experiments were designed to systematically vary key features of the original victim framing scenario: the source of the victim label (self vs. third party; Experiment 1); the gender and sexual orientation of the protagonists (Experiments 2 and 3); the type of misconduct and social status of the alleged perpetrator (Experiments 4 and 5); and whether the facts of the case are under dispute (Experiment 5). We highlight that the first three experiments focused on sexual assault because this is a domain where victim framing is frequently deployed in public discourse (and in the original work on this topic). Varying the gender dynamics within this domain allowed us to test whether the effects depend on cultural scripts about prototypical victims of sexual assault. Experiments 4 and 5 then extended this paradigm to different scenarios (physical domestic abuse and police shooting) to assess broader generalizability.

A footnote to explain the elements of the Dark Triad elements would be useful…

We appreciate this suggestion. Because PLOS ONE does not allow footnotes, we have added some additional explanation at first mention in the main text of the Experiment 1 methods (lines 252-254), noting that the Dark Triad refers to three socially aversive personality traits: (1) Machiavellianism (strategic manipulation); (2) psychopathy (callousness and lack of empathy); and (3) narcissism (grandiosity and entitlement). We agree that bringing this into the text is worthwhile.

…as would a paragraph outlining the limitations of the study. Currently the limitations are in the final section of the paper, but I would suggest bringing this into the methodology instead, rather than presenting some fantastic findings only to leave the readers’ last thoughts about the limitations of them.

We appreciate the spirit of this suggestion, which we understand as a concern that readers should be aware of methodological constraints before encountering the findings. However, in our experience it is standard practice in experimental psychology to present limitations in the General discussion, where they can be evaluated in light of the actual results. Placing a limitations section within the Methods, before the findings have been presented, risks priming readers to discount the results before they have had an opportunity to evaluate them. To address the concern about ending the manuscript with limitations, we have expanded the final part of the General discussion (now under the subheading “Conclusions and future directions”) to highlight the theoretical and practical implications of the findings and show how the limitations of the work motivate several exciting future directions. We believe that these additions will enable readers to better evaluate the limitations alongside the contributions of the findings.

The results and discussion section begins with a number of technical terms which require a short, simple explanation to make them accessible to a non-technical audience (JASP, ANOVA).

We have added brief parenthetical definitions of these terms at first use. JASP is now introduced as “an open-source statistical analysis program,” and ANOVA is now introduced as “analyses of variance (ANOVAs)—standard statistical tests for comparing group means.” We have also expanded other technical terms at first use to improve accessibility.

Given the huge amount of data collected and analysed, there is evidently a lot to discuss and there is a lot of evidence provided in the results section. At times this can be difficult to follow. I would encourage the authors to include a headline summary or insert box directly under the heading of each experiment in this section to make it really clear for the audience to understand what the most significant findings were in each experiment.

We appreciate this suggestion. We took care in our revision to be clearer about the key findings of each experiment in the experiment-specific Discussion subsections of the Results and Discussion section. At the suggestion of Reviewer 2, we have also added a new figure to the General discussion (Fig 6) that depicts overall effect sizes for citers and nonciters in each experiment, which provides an accessible visual representation of our central findings. Additionally, we have added a sentence to the figure caption for each experiment (Figs 1-5) that summarizes the pattern of results for citers and nonciters.

Expand acronyms SE when first used.

Done. SE is now written as “standard errors (SEs)” at first use (line 402) and in the table notes.

When discussing experiment 4, it would be clearer to use the surname of the relevant ‘Nick’, as currently it is difficult to determine whether we are reading about the plumber or the celebrity in each mention of ‘Nick’.

We appreciate this concern, but we believe the text already mostly addresses it: the first mention of “Nick” in the Experiment 4 results section explicitly identifies him as “Nick (Cage/Hatch), the alleged perpetrator,” establishing that “Nick” refers to the generic male character across both celebrity status conditions. All subsequent uses of “Nick” follow from this introduction, and we have reviewed the section to confirm there is no ambiguity (i.e., we never use “Nick” to refer to one individual in particular, the plumber or celebrity). That said, we have also made one additional clarification that should help readers keep track of which last names belong to which characters (lines 686-688): “Overall, participants supported Park more when she accused a stranger (Hatch) of assault than when she accused a celebrity (Cage), and supported Nick more when he was a celebrity than when he was a stranger.” (parentheses added in revised manuscript).

Line 609 ‘Overall, participants Park more when she accused a stranger…’ – There is a word missing here.

Good catch, thank you. The sentence has been fixed so it now reads “Overall, participants supported Park more when she accused a stranger…”

In the discussion relating to accusing celebrities, there are some suggestions that this could indicate people may question motivations of women who accuse celebrities of crimes. Some references to other sources that discuss this here would help to support this claim.

We appreciate this suggestion. In the updated manuscript, we have added several references to relevant literature on this point. Specifically, we now cite the following additional sources in both the Experiment 4 methods and results and discussion sections:

Knight, J. L., Giuliano, T. A., & Sanchez-Ross, M. G. (2001). Famous or infamous? The influence of celebrity status and race on perceptions of responsibility for rape. Basic and Applied Social Psychology, 23(3), 183–190. https://doi.org/10.1207/S15324834BASP2303_4

Nyúl, B., Kende, A., Engyel, M., & Szabó, M. (2018). Perception of a perpetrator as a successful person predicts decreased moral judgment of a rape case and labeling it as rape. Frontiers in Psychology, 9, 2555. https://doi.org/10.3389/fpsyg.2018.02555

Meyer, A. (2025). Blaming the victim, preserving the icon: the gendered moral work of celebrity sexual abuse scandals. Feminist Media Studies, 25(8), 1890–1904. https://doi.org/10.1080/14680777.2024.2392102

There are surprisingly few references to extant literature in the results and discussion. Some references which indicate how the findings support or challenge current understandings for each experiment would strengthen this section.

We agree that the experiment-specific Discussion subsections were relatively light on connections to the broader literature. In the revised manuscript, we have added references throughout these sections to make these connections clearer. Specifically, in Experiment 1 we now connect the finding that self- and other-framing are equally effective in this context to the literature on Truth-Default Theory (Levine, 2014). In Experiment 2, we reference work on male victimization and the resistance to recognizing men as victims/patients (Bullock & Beckson, 2011; Reynolds et al., 2020). In Experiment 3, we now cite research on attitudes towards same-sex intimate partner violence (e.g., Rolle et al., 2018). In Experiment 4, we connect the findings to the research cited above showing that fame predicts decreased responsibility attributions and moral condemnation in rape cases (Knight et al., 2001; Nyúl et al., 2018). And in Experiment 5, we highlight connections to research on how linguistic framing and ideological orientation shape evaluations of police violence (Gerber & Jackson, 2017; Park et al., 2024)

Perhaps the ‘General Discussion’ heading would be better renamed to ‘Conclusion’ given that there is already a ‘Results and Discussion’ section.

We appreciate this suggestion but prefer to retain “General discussion” because this section includes substantial interpretative and theoretical discussion in addition to summarizing key findings. As mentioned above, we have added a “Conclusions and future directions” subsection to the General discussion that summarizes our overall conclusions. To add further clarity, we have also added a specific “Limitations” subsection to the General discussion. The “Results and discussion” section is an umbrella heading for the experiment-specific results subsections. In our experience, this organization is common in experimental psychology.

In the general discussion, experiment 5 is described as a racially-charged police shooting. This racially-charged element does not come across in earlier mentions of experiment 5. The authors need to add clarity as to the racial element here. Was it a mistake to include it in the general discussion, or was it racially framed? If the latter, this needs mentioning in the methods and results of experiment 5, and consideration given to whether race plays a role in the readers’ responses.

This is an important point that we should have flagged more clearly. The names of the protagonists in Experiment 5 (Jamal Smith vs. John O’Neil) were deliberately chosen to evoke racial associations commonly present in media coverage of police violence, consistent with then-recent public discourse about White officers using force against Black civilians. However, given that we did not explicitly manipulate race or measure racial perceptions, we are careful about making strong claims. In the revised manuscript, we now preview this dimension of the experiment in the Introduction (lines 128-131) and have added several sentences to the Experiment 5 Methods section noting that the names were selected to evoke the racial dynamics commonly associated with publicized incidents of police violence, while acknowledging that we did not directly manipulate or measure participants’ racial attributions (Lines 350-357). We have also slightly revised the General discussion to refer to the “implicitly” racially-charged nature of the scenario rather than presenting it as a definitive racial manipulation (Lines 827-829). This is consistent with the more detailed treatment of this point in the Supplemental Material (S1 Text; Experiment 5 pilot study section).

There is a really interesting discussion of the age and political persuasion of the participants and how these might influence their responses. Adding references to wider literature that discusses how these elements may influence individuals’ approaches to crimes and the parties involve would be valuable and relevant.

Thank you for the suggestion. We have now added the following references to this part of the General Discussion, citing (a) research on the relationship between political ideology and attitudes towards crime and punishment, and (b) generational differences in attitudes towards sexual assault and consent (line 857).

Nagel, B., Matsuo, H., McIntyre, K. P., & Morrison, N. (2005). Attitudes toward victims of rape: Effects of gender, race, religion, and social class. Journal of Interpersonal Violence, 20(6), 725–737. https://doi.org/10.1177/08862605052760

Silver, J. R., & Silver, E. (2017). Why are conservatives more punitive than liberals? A moral foundations approach. Law and Human Be

Attachment Submitted filename: Response_to_Reviewers.docx

# Peer review: Decision Letter 1

**DOI:** 10.1371/journal.pone.0351416.r003

**Authors:** Annesha Sil

27 May 2026

Victim framing shapes attitudes across diverse contexts

PONE-D-25-65711R1

Dear Dr. Flusberg,

We’re pleased to inform you that your manuscript has been judged scientifically suitable for publication and will be formally accepted for publication once it meets all outstanding technical requirements.

Within one week, you’ll receive an e-mail detailing the required amendments. When these have been addressed, you’ll receive a formal acceptance letter and your manuscript will be scheduled for publication.

An invoice will be generated when your article is formally accepted. Please note, if your institution has a publishing partnership with PLOS and your article meets the relevant criteria, all or part of your publication costs will be covered. Please make sure your user information is up-to-date by logging into Editorial Manager at Editorial Manager® and clicking the ‘Update My Information' link at the top of the page. For questions related to billing, please contact billing support.

If your institution or institutions have a press office, please notify them about your upcoming paper to help maximize its impact. If they’ll be preparing press materials, please inform our press team as soon as possible -- no later than 48 hours after receiving the formal acceptance. Your manuscript will remain under strict press embargo until 2 pm Eastern Time on the date of publication. For more information, please contact onepress@plos.org.

Kind regards,

Annesha Sil, Ph.D.

Staff Editor

PLOS One

Additional Editor Comments (optional):

Reviewers' comments:

Reviewer's Responses to Questions

**Comments to the Author**

1. If the authors have adequately addressed your comments raised in a previous round of review and you feel that this manuscript is now acceptable for publication, you may indicate that here to bypass the “Comments to the Author” section, enter your conflict of interest statement in the “Confidential to Editor” section, and submit your "Accept" recommendation.

Reviewer #1: All comments have been addressed

Reviewer #2: All comments have been addressed

**********

2. Is the manuscript technically sound, and do the data support the conclusions?

The manuscript must describe a technically sound piece of scientific research with data that supports the conclusions. Experiments must have been conducted rigorously, with appropriate controls, replication, and sample sizes. The conclusions must be drawn appropriately based on the data presented.

Reviewer #1: (No Response)

Reviewer #2: Yes

**********

3. Has the statistical analysis been performed appropriately and rigorously?

Reviewer #1: (No Response)

Reviewer #2: Yes

**********

4. Have the authors made all data underlying the findings in their manuscript fully available?

The PLOS Data policy requires authors to make all data underlying the findings described in their manuscript fully available without restriction, with rare exception (please refer to the Data Availability Statement in the manuscript PDF file). The data should be provided as part of the manuscript or its supporting information, or deposited to a public repository. For example, in addition to summary statistics, the data points behind means, medians and variance measures should be available. If there are restrictions on publicly sharing data—e.g. participant privacy or use of data from a third party—those must be specified.

Reviewer #1: (No Response)

Reviewer #2: Yes

**********

5. Is the manuscript presented in an intelligible fashion and written in standard English?

PLOS ONE does not copyedit accepted manuscripts, so the language in submitted articles must be clear, correct, and unambiguous. Any typographical or grammatical errors should be corrected at revision, so please note any specific errors here.

Reviewer #1: (No Response)

Reviewer #2: Yes

**********

6. Review Comments to the Author

Please use the space provided to explain your answers to the questions above. You may also include additional comments for the author, including concerns about dual publication, research ethics, or publication ethics. (Please upload your review as an attachment if it exceeds 20,000 characters)

Reviewer #1: (No Response)

Reviewer #2: The study is quite promising after the revision. The authors addressed all queries, and the manuscript is now publishable. I recommend acceptance.

**********

7. PLOS authors have the option to publish the peer review history of their article (what does this mean?). If published, this will include your full peer review and any attached files.

If you choose “no”, your identity will remain anonymous but your review may still be made public.

**Do you want your identity to be public for this peer review?** For information about this choice, including consent withdrawal, please see our Privacy Policy.

Reviewer #1: No

Reviewer #2: No

**********

# Editor report: Acceptance letter

**DOI:** 10.1371/journal.pone.0351416.r004

**Authors:** Annesha Sil

PONE-D-25-65711R1

PLOS One

Dear Dr. Flusberg,

I'm pleased to inform you that your manuscript has been deemed suitable for publication in PLOS One. Congratulations! Your manuscript is now being handed over to our production team.

At this stage, our production department will prepare your paper for publication. This includes ensuring the following:

* All references, tables, and figures are properly cited

* All relevant supporting information is included in the manuscript submission,

* There are no issues that prevent the paper from being properly typeset

You will receive further instructions from the production team, including instructions on how to review your proof when it is ready. Please keep in mind that we are working through a large volume of accepted articles, so please give us a few days to review your paper and let you know the next and final steps.

Lastly, if your institution or institutions have a press office, please let them know about your upcoming paper now to help maximize its impact. If they'll be preparing press materials, please inform our press team within the next 48 hours. Your manuscript will remain under strict press embargo until 2 pm Eastern Time on the date of publication. For more information, please contact onepress@plos.org.

You will receive an invoice from PLOS for your publication fee after your manuscript has reached the completed accept phase. If you receive an email requesting payment before acceptance or for any other service, this may be a phishing scheme. Learn how to identify phishing emails and protect your accounts at https://explore.plos.org/phishing.

If we can help with anything else, please email us at customercare@plos.org.

Thank you for submitting your work to PLOS ONE and supporting open access.

Kind regards,

PLOS ONE Editorial Office Staff

on behalf of

Dr Annesha Sil

Staff Editor

PLOS One
